# Bioinspired Soft Elastic Metamaterials for Reconstruction of Natural Hearing

**DOI:** 10.1002/advs.202207273

**Published:** 2023-04-28

**Authors:** Hanchuan Tang, Shujie Zhang, Ye Tian, Tianyu Kang, Cheng Zhou, Shuaikang Yang, Ying Liu, Xurui Liu, Qicai Chen, Hongjun Xiao, Wei Chen, Jianfeng Zang

**Affiliations:** ^1^ School of Integrated Circuits and Wuhan National Laboratory for Optoelectronics Huazhong University of Science and Technology Wuhan 430074 China; ^2^ College of Life Science and Technology Huazhong University of Science and Technology Wuhan 430074 China; ^3^ School of Life Sciences Central China Normal University Wuhan 430074 China; ^4^ Department of Otorhinolaryngology Union Hospital Tongji Medical College Huazhong University of Science and Technology Wuhan 430022 China; ^5^ The State Key Laboratory of Digital Manufacturing Equipment and Technology Huazhong University of Science and Technology Wuhan 430074 China

**Keywords:** cochlear implants, elastic metamaterials, natural hearing, piezoelectric materials, soft materials

## Abstract

Natural hearing which means hearing naturally like normal people is critical for patients with hearing loss to participate in life. Cochlear implants have enabled numerous severe hearing loss patients to hear voice functionally, while cochlear implant users can hardly distinguish different tones or appreciate music subject to the absence of rate coding and insufficient frequency channels. Here a bioinspired soft elastic metamaterial that reproduces the shape and key functions of the human cochlea is reported. Inspired by human cochlea, the metamaterials are designed to possess graded microstructures with high effective refractive index distributed on a spiral shape to implement position‐related frequency demultiplexing, passive sound enhancements of 10 times, and high‐speed parallel processing of 168‐channel sound/piezoelectric signals. Besides, it is demonstrated that natural hearing artificial cochlea has fine frequency resolution up to 30 Hz, a wide audible range from 150–12 000 Hz, and a considerable output voltage that can activate the auditory pathway in mice. This work blazes a promising trail for reconstruction of natural hearing in patients with severe hearing loss.

## Graphical abstract

A bioinspired soft elastic metamaterial with a size equivalent to the current cochlear implant that can be seen as a prototype of passive artificial cochlea with a frequency resolution of 30 Hz is proposed. This work will inspire future work in the fabrication of totally implantable passive artificial cochlea that enables the users to hear naturally.

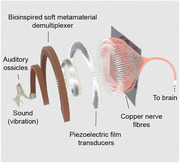

## 1 Introduction

**Figure 1:**
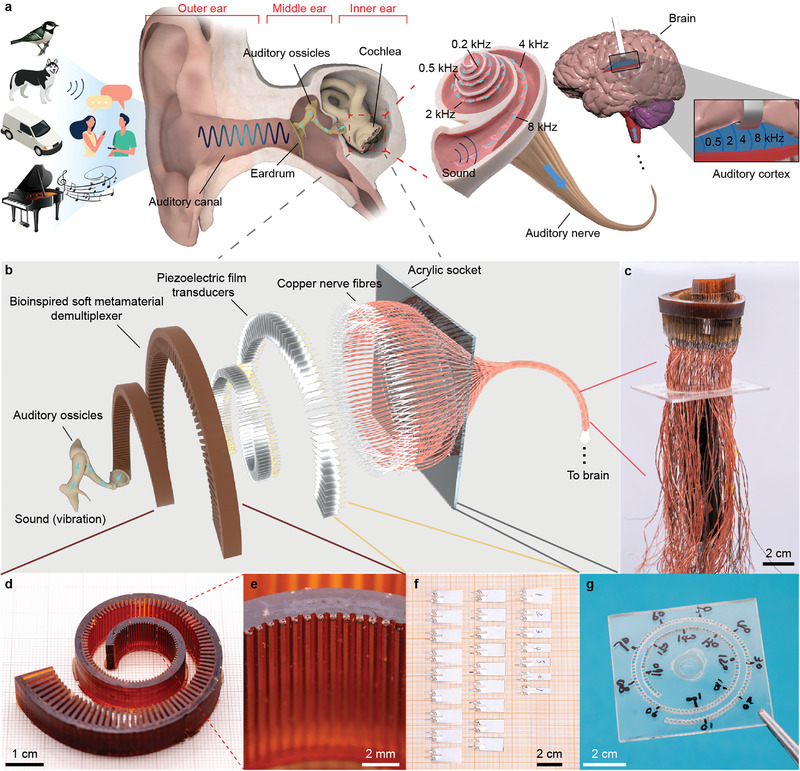
Human natural hearing process and the design of BSEM. a) Natural hearing process of normal people. External sounds are transmitted to the brain through the outer ear, middle ear, inner ear, and auditory nerve pathway. b) The detailed structure of BSEM. BSEM can replace the defective cochlea to bridge the auditory pathway. c) Side view of a completed BSEM. d,e) Photographs of the metamaterials sample (without piezoelectric flakes) and enlarged branch microstructures, respectively. f,g) Photographs of piezoelectric flakes and acrylic sockets, respectively. The animal, car, piano, and people icons are designed by Freepik.

In the natural hearing process of normal people (**Figure** **1**a), external sounds including speech, music, and different tones are collected by the external ear and stimulate the eardrum. Then the sounds (vibrations) propagate to the cochlea through the filtering and amplification (including active and passive) of auditory ossicles. In the cochlea, each specific frequency of sound activates a corresponding specific group of hair cells at a specific place, which is called place coding. Meanwhile, each hair cell is triggered synchronizing the real‐time changes of external sound (mainly mid‐to‐low frequencies), which is called rate coding. Thus, sound signals are transmitted to auditory nerves through hair cells in the cochlea and then to the corresponding regions in the auditory cortex through auditory nerves.^[^ ^1^ ^]^ However, globally 1/5 of people are suffering from some degree of hearing loss, estimated 430 million of whom belong to moderate or higher severity.^[^ ^2^ ^]^ The deficiency of hair cells is the main reason for severe or profound hearing loss.

Cochlear implants (CI), which have been commercialized for nearly 40 years, brought functional hearing to numerous hearing loss patients by generating electric currents to directly stimulate the auditory nerve. The route of CI is to stimulate auditory nerves with implanted current sources and electrodes while ignoring the users’ original auditory path. CI users usually have adequate communication abilities in a relatively quiet environment but can hardly distinguish different tones, recognize speech signals from noises, or appreciate music mainly subject to the absence of rate coding and insufficient frequency resolution.^[^ ^3^, ^4^ ^]^ Hearing naturally like normal people is a long‐term wish of patients with severe or profound hearing loss, but has not been implemented.^[^ ^5^ ^]^

To improve the auditory performance of CI, efforts were made by increasing frequency channels and adding partial temporal fine structure into the stimulating signal.^[^ ^6^ ^]^ Nevertheless, either increasing frequency channels or providing variable‐frequency stimuli will rapidly increase both the power consumption and the size of the CI. Therefore, the average frequency resolution of current CI is about 300 Hz in 0–8 kHz, while the frequency variation between two pitches in music can be less than 30 Hz. As for other aspects of CI, totally implantable cochlear implants^[^ ^7^, ^8^ ^]^ were investigated by implanting the external devices of CI into human body to avoid discrimination. Besides, artificial basilar membranes or hair cells^[^ ^9^, ^10^, ^11^, ^12^ ^]^ were explored to obtain a passive sound‐electric response to simplify the structure of CI. But good pitch‐place mapping with high frequency resolution has not yet been achieved. Acoustic metamaterial^[^ ^13^ ^]^ is one of the prominent technologies for manipulating sound waves at will. Previous works about acoustic metamaterials with effective refractive index ^[^ ^14^, ^15^, ^16^, ^17^ ^]^ demonstrated pitch‐place mapped acoustic concentrators at relatively high frequencies (e.g., 3–10 kHz) with tens of centimeters in size. If the working frequency comes to hundreds of hertz, the sample size will be several meters. Several previous studies on metamaterials^[^ ^18^, ^19^, ^20^, ^21^ ^]^ have explored cochlea‐inspired acoustic sensors or cochlea‐like vibration modes in rigid resonators. However, few have demonstrated hearing reconstruction, acoustic‐electric transversion, or biomedical engineering applications. Acoustic metamaterials give promising opportunities for implementing pitch‐place mapping and sound‐electric conversion in cochlea but are limited to the bulky and rigid body.

Here we report a bioinspired soft elastic metamaterial (BSEM) that reproduces the shape and function of the human cochlea. BSEM keeps nearly the same auditory path as normal people and reproduces the key functions of human cochlea, as shown in Figure S1 (Supporting Information). Through utilizing soft materials as matrix and reasonable structure design of metamaterials, the whole size of BSEM can be reduced to several centimeters, with up to 168 frequency channels that cover the audible range of 150–12 000 Hz. Besides, the bioinspired soft elastic metamaterial can be stretched or bent to fit the spiral canal in the cochlea while maintaining good pitch‐place mapping performance. Then we verified that BSEM can distinguish different pitches in C major scale by theoretical analysis, simulations, and experiments. Animal experiments indicate that BSEM can activate the auditory pathway in mice without a power source.

## 2 Results

A detailed structure of BSEM is shown in Figure 1b. As a comparison, a typical modern cochlear implant system is mainly composed of external devices (microphone, processor, transmitter, etc.) and implants (receiver, stimulator, spiral wires, etc.), as shown in Figure S2 (Supporting Information). The bioinspired soft elastic metamaterial serves as an energy concentrator as well as a demultiplexer to concentrate and enhance the sound at different branches in BSEM according to its frequency when the sound naturally propagates to the cochlea from auditory ossicles. A piezoelectric flake inserted in each branch of the matrix serves as an independent mechanical‐electro converter. The copper wires work as the media to transmit electric signals into the auditory nerves or cortex. An acrylic socket was utilized to distinguish different channels. Each sound‐electric channel can be addressed and measured by selecting the corresponding copper wire. The working mechanism of BSEM resembles that of the human cochlea, in which groups of hair cells are distributed below different frequency‐responsive regions of the basilar membrane and individually connected with auditory nerve fibers,^[^ ^22^ ^]^ enabling fine place‐pitch mapping and high‐speed parallel processing of electric signals. Moreover, the whole process is analog, which means the electric signals converted by BSEM can synchronize the change of external sound (complete rate coding) without extra delay compared to the natural hearing process. The side view of a completed BSEM is exhibited in Figure 1c. The detailed fabrication process of BSEM is described in Methods and Figure S3 (Supporting Information). Figure 1d,e exhibits the photographs of the metamaterial sample and branch microstructure (without piezoelectric flakes), respectively. The metamaterial is made of a urethane rubber (PMC 780, smooth‐on) by reverse mold. The density, Young modulus, and Poisson's ratio of PMC 780 are 1020 kg m^−3^, 1.69 MPa (measured by uniaxial tensile tests, Figure S4, Supporting Information), and 0.49, respectively. Figure 1f,g shows photographs of piezoelectric flakes and acrylic sockets. The piezoelectric flakes with varying sizes comprise a 28 µm thick polyvinylidene difluoride (PVDF) flake with screen‐printed silver ink electrodes, laminated to a 0.125 mm polyester substrate (LDT0‐028K, TE connectivity). The density, Young modulus, and Poisson's ratio of PVDF are 1780 kg m^−3^, 2 GPa, and 0.35, respectively.

**Figure 2:**
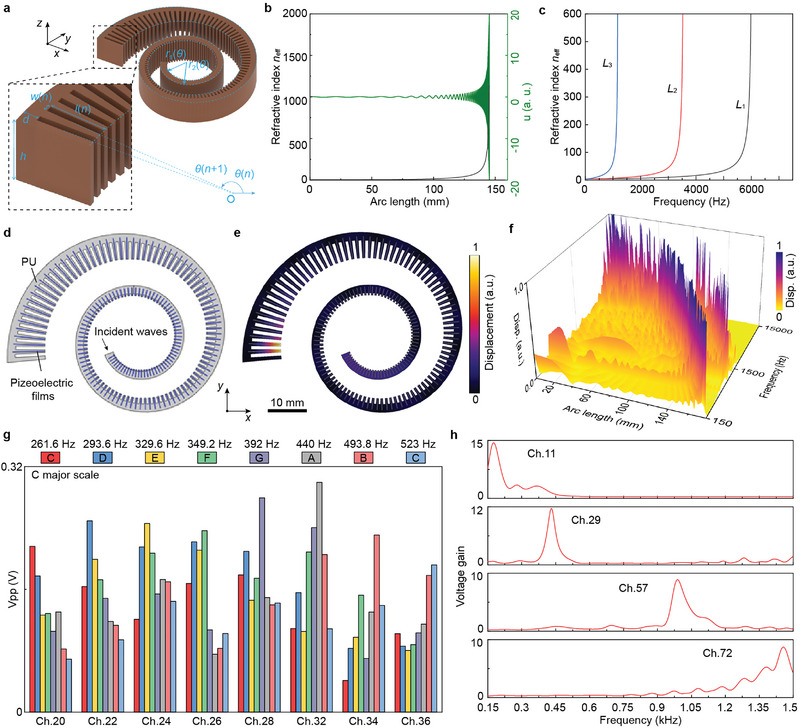
Theoretical analysis, simulation, and experimental characterization of rate coding and place coding. a) Schematic of a bioinspired soft elastic metamaterial. b) Calculated effective index and vibration distribution along the arc length *L* of the bioinspired metamaterial. The wave compression effect is observed accompanied by an increase in the effective index. c) Dispersion properties of the effective index at different locations along the propagation axis. d) A simulation model for metamaterials with piezoelectric flakes. e) Simulated displacement distribution in metamaterials when elastic waves are incident at 150 Hz. f) Simulated place‐pitch mapping of BSEM. g) Peak to peak voltage (*V*_pp_) of 8 channels in BSEM under tone‐burst excitations in C major scale. The excitation intensity is calibrated as the sound with a loudness of 70 dB. h) Voltage gains of 4 channels in BSEM by comparing the output voltage of an unstructured sample.

The core of our method for rehabilitating natural hearing is the bioinspired metamaterials, a kind of soft elastic metamaterials^[^ ^23^, ^24^, ^25^ ^]^ with a bioinspired deformable spiral body and frequency‐related high effective refractive index in each branch. The soft spiral body allows the metamaterial to be inserted into the cochlea with little damage to human. The high effective refractive index enables a spatial concentration of wave energy and induces a strong pressure enhancement in the branches of the metamaterial.^[^ ^14^, ^15^, ^16^, ^26^ ^]^ The local effective refractive index of metamaterial is meticulously designed to obtain a place‐pitch mapping in accordance with human audiological characteristics with both a wide audible range and enough frequency resolution. **Figure** **2**a exhibits a typical design of BSEM. BSEM possesses a spiral base with branches of varying lengths. The overall spiral shape of BSEM is described by two curves *r*_1_(*θ*) = 1.8 × 1.09*^*θ*^* mm and *r*_2_(*θ*) = *r*_1_(*θ*) + *d*(*θ*) + *w*(*θ*), (6.01*π* < *θ* < 9.91*π*). The branches are formed by cutting minimal cuboids from the ensemble spiral tapes. The length, width, and angular position of cut cuboids are *l*(*n*) = 2.4(168−*N*)^7^ × 10^−17^ mm + 0.27 mm, *w*(*N*) = 0.1 × 1.0067^169−N^ mm, *θ*(*N*) = 0.255*π*[(169−*N*)^0.64^−1], (*N* = 1, 2, 3, …, 168), respectively. The bottom margin *d* and height of BSEM are 2.1 mm and 2.0 cm, respectively.

The whole structure of the metamaterial can be seen as an equivalent material, as shown in Figure S5 (Supporting Information). According to the designed structure and equivalent medium theory (Supporting Information), we theoretically calculated the effective refractive index of BSEM when it guides a 200 Hz acoustic wave (Figure 2b). The effective refractive index increases from 3.84 (PMC 780) to a very large value of 2000 at arc length *L* = 142.8 mm (total length of BSEM is 219.7 mm) along the propagation direction. At the same time, the wavelength gradually decreases and the wave pressure field is progressively growing, which renders a spatial compression of the acoustic wave as well as the amplitude enhancement in the high refractive index region. Besides, the effective refractive index for different frequencies will reach a maximum value at different locations (For instance *L*_1_ = 8.2 mm, *L*_2_ = 33.5 mm, and *L*_3_ = 105 mm correspond to 6 kHz, 3.5 kHz, and 1.2 kHz, respectively, as shown in Figure 2c). Thus, elastic waves will be enhanced at different positions from the first branch to the last of BSEM when the frequency decreases.

The above analysis didn't take into account the influence of some realistic factors like the vibration loss in soft materials and the addition of piezoelectric flakes. Here we conducted finite element simulations to further verify the effects of BSEM. A simulation model for BSEM with piezoelectric flakes inserted in each branch is depicted in Figure 2d. When elastic waves at 150 Hz are incident from the small side of BSEM, most vibration energy will concentrate at the 167th branch (Figure 2e). The whole displacement distributions of elastic waves with different frequencies from 150 Hz to 12 kHz are shown in Movie S1 (Supporting Information), in which elastic waves were enhanced at different positions from the first branch to the 168th of BSEM. Figure 2f exhibits the complete place‐pitch mapping of BSEM, where the displacement peaks constitute a power‐law envelope. Meanwhile, we considered two other models including BSEM without piezoelectric flakes (the control group A) and unstructured matrix with piezoelectric flakes (the control group B) to illustrate the characteristics of BSEM. The simulation model and boundary condition settings are shown in Figure S6 (Supporting Information). Displacement distributions of three models at different frequencies are depicted in Figure S7 (Supporting Information). Compared to BSEM without piezoelectric flakes, the vibration‐enhanced of BSEM at the same frequency is shifted by several branches. In the control group B, elastic waves are reflected and form a standing wave. These effects are clear by analyzing the displacement distribution along the angular direction of three models (Figure S8, Supporting Information).

The above analyses are based on one typical design of BSEM. The structural parameters of BSEM can be adjusted to fit different situations. The overall shape is designed to be a kind of logarithmic spiral, just like the shape of human cochlea. Actually, the place‐pitch mapping is mainly determined by the gradient effective refractive index design, i.e., the branch length. The overall shape has very little influence on the concentration effect in our design, which means our design allows the change of overall shape when keeping the morphology of branches. Besides, the influence of bottom margin *d* was analyzed (Figure S9, Supporting Information). The simulated results show that minor adjustments of *d* have a small impact on the concentration effect of BSEM. We also conducted a simulation to illustrate the influence of the width of each branch, as shown in Figure S10 (Supporting Information). The results show that the position of the vibration maximum has changed a little when the width varies. The width of each branch influences the filling ratio of air. According to Equation (S6) (Supporting Information), the contribution of the filling ratio to the effective refractive index is limited. Therefore, the width was not utilized as the key parameter in the design. The width we utilized in the manuscript keeps the filling ratio being near 0.5 for the convenience of fabrication. As for the scale effect, when the overall size of BSEM is reduced or increased, the material selection requires reconfiguration to maintain the original working frequencies according to Equation (S4) (Supporting Information).

After fabricating the BSEM sample according to the optimized structure, a biomimetic measuring system was built to evaluate the performance of BSEM (Figure S11, Supporting Information). Most of the 168 channels were measured at different excitation conditions. Figure 2g illustrates the peak‐to‐peak voltage (*V*_pp_) of 8 channels (20th, 22nd, 24th, 26th, 28th, 32nd, 34th, 36th) in BSEM at a series of tone‐burst excitations (central frequencies are in the C major scale, duration is ≈40 ms). The results show good pitch discrimination and considerable frequency resolution (≈30 Hz) of BSEM. We also tested the performance of BSEM at a broad frequency range. The *V*_pp_ of 8 channels (3rd, 8th, 14th, 33rd, 49th, 57th, 77th, 90th) in BSEM at a series of tone‐burst excitations (central frequencies are from 120 to 3000 Hz, duration is ≈40 ms) are illustrated in Figure S12 (Supporting Information). The maximum testing frequency is set to 3000 Hz due to the limit of the exciter. The output voltage decreases with the increase of vibration propagation distance due to the loss of soft materials, while the results indicate frequency selectivity in each channel. In general, BSEM has a wide audible range and enough frequency resolution.

**Figure 3:**
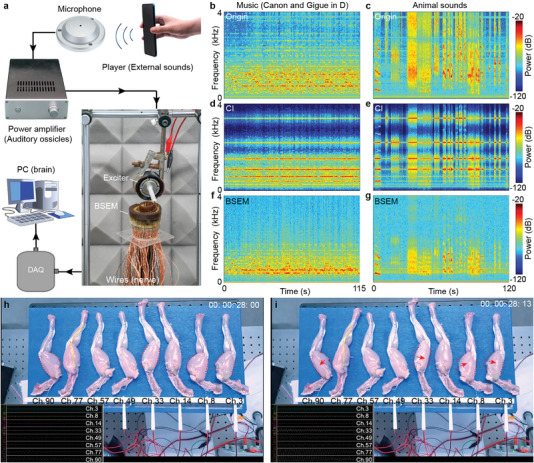
Demonstration for distinguishing different tones and appreciating music. a) Experimental setup to simulate the hearing process of human. b,c) Original spectrograms of a piano song, and animal sounds, respectively. The frequency range is limited to 0–4 kHz. d,e) Spectrograms (processed by an 8‐channel CI) of a piano song, and animal sounds, respectively. f,g) Spectrograms (processed by 8 channels of BSEM) of a piano song, and animal sounds, respectively. h,i) Two frames in Movie S9 (Supporting Information). Isolated hind limbs repeated bouncing synchronizing amplified electrical stimulations from BSEM.

In order to evaluate the voltage enhancement effect of metamaterials, we prepared a sample without microstructure. Eight (3rd, 11th, 29th, 57th, 72nd, 102nd, 126th, 152nd) of 168 channels were picked and analyzed (Figure S13, Supporting Information). For the unstructured sample, measured voltage shows a decreasing trend when the wave propagation distance increases (i.e., channel number becomes smaller). As for the BSEM, the voltage of the 57th channel is the maximum among these channels for an excitation frequency of 420 Hz. We further use voltage gain (obtained by *V*_BSEM_(*f*)/*V*_unstructured_(*f*), *V* and *f* for voltage and frequency respectively) to characterize the frequency response of each channel. *V* (*f*) is obtained by Fourier transform of the output voltage of samples under sweep excitation. **Figure** **3**h shows the voltage gain of four channels (11th, 29th, 57th, and 72nd), where voltage gain peaks are at 200 Hz, 430 Hz, 1 kHz, and 1.46 kHz, respectively. The average voltage gain is over 10 times.

In addition, we conducted more tests to comprehensively evaluate BSEM. The voltage signals produced by single short‐time excitation (Figure S14, Supporting Information) indicate that there is hardly any delay (20 ms, which mainly comes from the delay of exciter) between the beginning of excitation and the generation of the vibration‐electrical signal. Meanwhile, there is a small natural delay (several milliseconds) among the different channels due to the propagation of elastic waves, which is consistent with the natural hearing process. Moreover, we test the vibration‐electrical response of BSEM under different deformations (Figure S15, Supporting Information). The change of spiral pitch will hardly cause the change of vibration‐electrical response in each channel, indicating good deformation tolerance of BSEM. The stability of BSEM under continuous tone‐burst testing is exhibited in Figure S16 (Supporting Information). The percentage standard deviation of the output voltage is mostly less than 4%.

After the characterization of BSEM, we conducted experiments to demonstrate the sound natural processing ability of BSEM by acquiring music and different tones. We utilized a player to play the sounds of music, different animals, different vehicles, and a conversation. The sounds recorded by a microphone and amplified by a power amplifier were utilized as signal sources to drive the exciter. The vibrations were converted into electronic signals by each channel of BSEM and recorded by a multichannel digital acquisition card. This resembles the natural hearing process, as shown in Figure 3a. At the same time, we collected the original sounds directly in the same environment and simulated the processing of CI. The processing methods of CI and BSEM are depicted in Figure S17 (Supporting Information). Thus the original sounds, sounds processed by CI, and sounds processed by BSEM were obtained. Figure 3b,c exhibits short‐time Fourier transform spectrograms of a piano song and animal sounds acquired in noisy environments. Figure 3d–g shows the spectrograms of sounds processed by CI and BSEM. The spectrograms processed by BSEM resemble the spectrograms of original sounds, while spectrograms processed by CI basically lose the fine frequency characteristics and only retain the fluctuation of intensity for a relatively long time. The results of more sounds (sounds of another song, different vehicles, and a conversation) are illustrated in Figure S18 (Supporting Information). Synthetic audios are shown in Movies S2–S5 (Supporting Information). Besides, we compare the similarity between original sounds and sounds processed by CI or BSEM based on their spectrograms (Figure S18, Supporting Information). Compared with the sounds processed by CI, the sounds processed by BSEM have a higher similarity with the original sounds.

Additionally, we conducted frog sciatic nerve stimulation experiments to further demonstrate BSEM. The experimental set‐up resembles Figure 3a, except that eight wires from different channels of BSEM were connected to eight stimulation electrodes. Sciatic nerves of eight frog legs were attached to stimulation electrodes, as depicted in Figure 3h. To make the frog leg flexion more obvious, the voltage of each channel was amplified (Charge amplification) before stimulating sciatic nerves. Concretely, stimulations were carried out under different sound sources (different pitches and piano songs), as shown in Movies S6–S9 (Supporting Information). Figures 3h,i are two frames intercepted in Movie S9 (Supporting Information). In the comparison of the two frames, frog muscles of channels 3, 8, 33, 77, and 90 contracted in varying degrees after one syllable. Subgraphs of Figure 3h,i exhibit the voltage change in each channel. More comparisons are illustrated in Figure S19 (Supporting Information). These in vitro experiments indicate fine place‐pitch mapping of BSEM.

**Figure 4:**
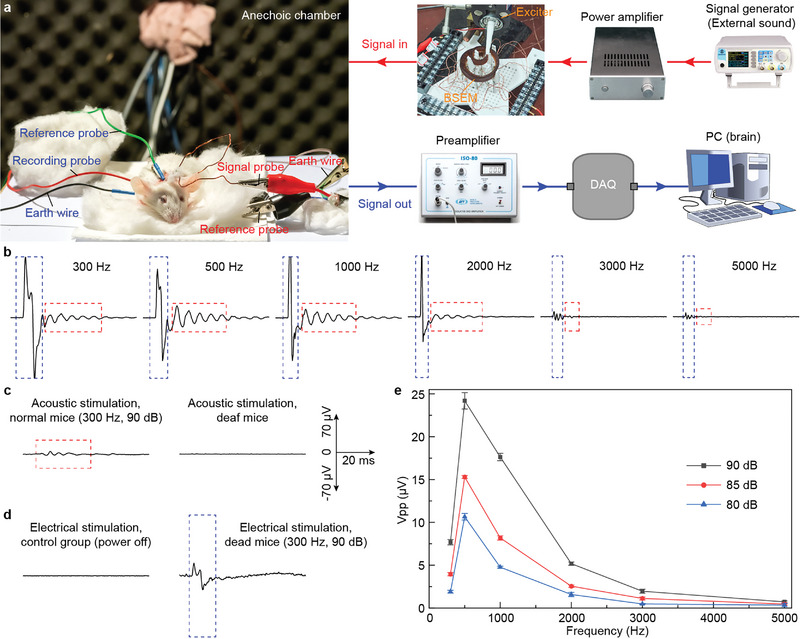
Validation for activation of auditory pathway by in vivo animal experiments. a) Experimental setup for ABR acquisition under direct electrical stimulations from BSEM. b) The ABR to tone bursts of 6 frequencies at direct electrical stimulations from BSEM. c) Sampling results with acoustic stimulation on normal mice and deaf mice (control). The right coordinates indicate the scale of the duration and amplitude of ABR in the whole figure. d) Results obtained when the cochlea was not stimulated at all, and obtained on deaf mice (control). e) The statistical results of ABR under direct electrical stimulations from BSEM at different frequencies. Each sampling is the summation and average of the evoked potentials induced by 300 electrical stimulation of the cochlea under each same stimulation condition, and the results were repeated 10 times.

In order to verify the biological effect of the electrical signal generated by BSEM, we stimulated auditory nerves in the cochlea of Kunming mice by inserting electrodes of BSEM and recorded their auditory brainstem response (ABR) that is often used to study and detect the functional state of the auditory system in basic and clinical practice.^[^ ^27^, ^28^, ^29^ ^]^ Part of the experimental set‐up is exhibited in **Figure** **4**a, where the mice are placed in the anechoic chamber to shield experiments from electromagnetic and acoustic interference. The insertion position of the electrode in mice is confirmed by optical and micro‐computed‐tomography images, as shown in Figure S20 (Supporting Information). Concretely, we conducted acoustic stimulation, electrical stimulation (with stimulators), and direct electrical stimulation. More details on the experimental process and set‐up are in Supporting Information and Figure S21 (Supporting Information). The results of direct electrical stimulation by BSEM are shown in Figure 4b and Figure S22 (Supporting Information) (different excitation intensities), which are similar to previous studies on human and animals.^[^ ^30^, ^31^, ^32^ ^]^ The results show that the electric signal with different frequencies (300–5000 Hz) and intensities (80–90 dB) converted by BSEM could lead to ABR in mice. For comparison, the results of normal mice and deaf mice (bilateral tympanic membrane and ossicular chain destroyed) under acoustic stimulations are illustrated in Figure 4c. ABR was observed in normal mice while no response was observed in deaf mice due to the interruption of sound propagation. Electric responses of dead mice (15 min after death) under the stimulation by BSEM (300 Hz, 90 dB) were also recorded (Figure 4d). The response became significantly weak, suggesting that the conductive characteristics of the body tissue^[^ ^33^ ^]^ were also undergoing irreversible changes. The mean peak‐to‐peak values (recorded 10 times) shown in Figure 4e indicate that the trend of response induced by each stimulation frequency is consistent with the change of stimulation amplitude. That is, the response decreases with the decrease of stimulation intensity at the same frequency; the intensity of response decreases with the increase of the converted acoustic stimulus frequency. The relationship between stimulus and response is the embodiment of the basic physiological characteristics of the cochlea. Meanwhile, the stimulus intensities for each frequency are recorded (Figure S23, Supporting Information). The stimulation intensity decreased significantly with the increase of frequency due to the limit of the exciter (low amplitude at high frequency) and attenuation of elastic waves (high attenuation at high frequency). The above results show that BSEM can be directly used to stimulate the cochlea to produce biological effects. More results about acoustic stimulations and electrical stimulations are depicted in Figures S24 and S25 (Supporting Information).

The above results indicate that the BSEM possesses a broad audible range, more than 150 frequency channels, a similar analog sound sensing mechanism to natural hearing, and good robustness. Besides, BSEM has the advantages of convenience (MRI‐compatible, battery‐free) and safety (immunity to electromagnetic interferences, good biocompatibility), which is hard to achieve for current electronic CI. Immunity to electromagnetic interferences and MRI‐compatibility are verified in Figures S26 (Movie S10) and S27 (Supporting Information), respectively.

## 3 Discussion

In this work, we provided a proof of concept for natural hearing artificial cochlea. The main results in the manuscript are obtained from a 168‐channels BSEM with the size of 5 cm × 5 cm (which is comparable with current 22‐channel CI, see Figure S2, Supporting Information). To fully implant BSEM, the size of BSEM needs to be further reduced. In a simulation (Figure S28, Supporting Information), we explored a configuration of 168‐channels BSEMs with the size of 5 mm × 4 mm, which is much smaller than the size of the current CI. We re‐established the material configuration and adjusted the microstructure to adapt to the cochlear internal environment. In this case, the matrix material of miniature BESM is replaced by a kind of hydrogel with the smaller shear modulus being 10 kPa and density being 1030 kg m^−3^. Air is sealed inside the matrix to keep body fluid out. We conducted simulations to predict the propagation of sound waves through small BSEM (Figure S7, Supporting Information). The results show that small BSEM possesses good place‐pitch mapping at different frequencies. However, the biggest challenge with miniature BSEM is fabrication. The miniature BSEM involves the manufacturing and assembly of soft polymers, metal electrodes, piezoelectric materials, and air. These materials with microstructures of ≈1‐µm are arranged in a specific order, which is difficult to integrate and form with current single manufacturing technology. Recent researches (relate to stereolithography^[^ ^34^ ^]^ and fiber drawing^[^ ^35^ ^]^ technologies) have shown the potential for manufacturing such complex microstructures. We will attempt to manufacture small BSEM based on these technical means in future work.

In conclusion, we proposed a design of BSEM which can be seen as a prototype of the passive artificial cochlea to achieve natural hearing. We described the principle of BSEM with theoretical analysis and simulations. Besides, we demonstrated the characteristics, convenience, and biological effects of BSEM by measurement and animal experiments. BSEM has up to 160 frequency channels that cover the audible range of 150 Hz ≈ 12 kHz with a passive analog output voltage up to 2 V. BSEM possesses a soft and deformable body with an integrated but concise structure. Meanwhile, the structure design of BSEM can be optimized for physiological place‐pitch mapping of each person for better performance. On the basis of this work, it is promising to prepare a totally implantable BSEM with better hearing experience, less inconvenience, and higher security, which could bring a natural hearing experience to people suffering from hearing loss.

## 4 Experimental Section

### Numerical Simulation

COMSOL Multiphysics 4.2a was used to conduct the finite element analysis for simulating the elastic waves propagating in SBMC. The type of the mesh element is free triangular and the maximum size is 0.3 mm (about *λ*/10) in the simulation. The linear elastic model (the isotropic damping is set to be 0.01) is utilized to simulate elastic waves propagating under different conditions. The simulation model and boundary condition settings are shown in Figure S6 (Supporting Information). For material parameters, many material configurations were examined in order to balance the enhancement, working frequency band, flexibility of the device, and sample size. Finally, a urethane rubber (PMC 780, smooth‐on) was chosen as the matrix. The mechanical property of PMC 780 by uniaxial tensile tests was obtained. First, two flat specimens with shoulders (used with serrated grips) were prepared (Figure S4a, Supporting Information). As shown in Figure S4b (Supporting Information), the data within the “small deform region” were picked out and used to establish a fitting profile. The slopes of fitting curves show Young's modulus of standard samples.

### Fabrication of BSEM

Molds with designed structures were printed by a 3‐dimensional printer (SprintRay Pro95, SprintRay Inc.). 168 shims with a thickness of 125 µm were inserted into the reserved gap in molds. The matrix materials (PMC 780, Smooth‐On Inc.) were poured into the molds after the release agent (Ease Release 200, Smooth‐On Inc.) was added to the inner surface of the mold. After being placed at 23 °C for 48 h, the samples were formed and taken out. Then the shims were replaced by piezoelectric flakes with the same thickness and similar density as well as modulus. Copper twisted pairs were welded to the electrodes of piezoelectric flakes. More details are shown in Figure S3 (Supporting Information).

### Measurement and Characterizing of BSEM

All the testing platforms were basically constructed with an optical bench, acoustic sponges, vibration exciters, amplifiers, and an 8‐channel data acquisition system (USB8584, ART Technology). Detailed measure setups for different experiments are shown in figures in main text and Supporting Information.

### Frog Leg Stimulation

Isolated hind limbs were obtained from frogs. Ringer's solution was added to isolated hind limbs to keep their physiological activity. In experiments, electrodes of 8 channels were attached to sciatic nerves in hind limbs for electrical stimulation.

### General Surgery of Mice

The animal experiments were approved by Institutional Animal Care and Use Committee, Huazhong University of Science and Technology with the IACUC number being 2714. A total of 31 healthy and normal‐hearing adult Kunming mice (Mus musculus, km) (40 ± 5 g; females) were used in the experiment. General surgery was performed after anesthesia with Nembutal sodium (45 mg kg^−1^, 1 wt%) intraperitoneal injection.^[^ ^29^, ^36^ ^]^ In brief, the hair on the top of the head and around one ear was cut, the skin on the head was cut with a scalpel to make a small incision and the local anesthetic procaine was dropped for pain relief, and the skull was exposed to fix the small flat nail (length is 10 mm, diameter is 1 mm) for fixing the cochlear stimulation electrode. Referring to previous relevant research reports,^[^ ^37^, ^38^ ^]^ the skin around one auricle was cut to expose the milky white translucent auditory bulla. A small hole was drilled in the auditory bulla with a probe, and part of the bone wall of the auditory bulla was peeled off along the hole with sharp‐nosed forceps to enlarge the opening for insertion of the stimulating electrode. Under a stereomicroscope, a small hole about 30–50 µm in diameter was drilled in the cochlear bone wall adjacent to the round or cochlear widow with a fine probe for inserting a thin insulted tungsten wire electrode for stimulating the cochlea, as shown in Figure S20 (Supporting Information).

## Conflict of Interest

H. T. and J. Z. are inventors of a patent application (CN patent application: 2022109096269) that covers the mechanism and the design of BSEM.

## Supporting information

Supporting Information

Supplemental Movie 1

Supplemental Movie 2

Supplemental Movie 3

Supplemental Movie 4

Supplemental Movie 5

Supplemental Movie 6

Supplemental Movie 7

Supplemental Movie 8

Supplemental Movie 9

Supplemental Movie 10

Supplemental Video 11

## Data Availability

The data that support the findings of this study are available from the corresponding author upon reasonable request.
