# Supplementary material for: Bioinspired Soft Elastic Metamaterials for Reconstruction of Natural Hearing
Source: Adv Sci (Weinh). 2023 Apr 28;10(20):2207273. doi: 10.1002/advs.202207273 (PMC10369269; doi:10.1002/advs.202207273)
Supplement: Supplementary file 1 — Supporting Information [file ADVS-10-2207273-s012.pdf]

ADVANCED SCIENCE
Open Access

Supporting Information

for *Adv. Sci.*, DOI 10.1002/advs.202207273

Bioinspired Soft Elastic Metamaterials for Reconstruction of Natural Hearing

Hanchuan Tang, Shujie Zhang, Ye Tian, Tianyu Kang, Cheng Zhou, Shuaikang Yang, Ying Liu, Xurui Liu, Qicai Chen, Hongjun Xiao, Wei Chen* and Jianfeng Zang**

Supporting Information

Bioinspired Soft Elastic Metamaterials for Reconstruction of Natural Hearing

Hanchuan Tang, Shujie Zhang, Ye Tian, Tianyu Kang, Cheng Zhou, Shuaikang Yang, Ying Liu, Xurui Liu, Qicai Chen, Hongjun Xiao, Wei Chen*, Jianfeng Zang**

Supporting Text

1. Calculation of the effective refractive index

The theory of effective refractive index can refer to ref. ***15*** and ref. ***16***. As shown in **Figure S5**, a two-dimensional (2D) system is considered, where the propagation of acoustic waves is confined in the θ-r plane. The entire metamaterial structure is equivalent to a continuous, lossless anisotropic metamaterial with effective mass density ρ_θ, ρ_r, and bulk modulus B, determined by the following effective medium model:

$$\rho_\theta = F\rho_{\mathrm{air}} + (1-F)\rho_{\mathrm{PMC}}, \tag{1}$$

$$\rho_r = \frac{\rho_{\mathrm{air}}\rho_{\mathrm{PMC}}}{(1-F)\rho_{\mathrm{air}} + F\rho_{\mathrm{PMC}}}, \tag{2}$$

$$B = \frac{B_{\mathrm{air}}B_{\mathrm{PMC}}}{(1-F)B_{\mathrm{air}} + FB_{\mathrm{PMC}}}. \tag{3}$$

where $F = w(N)/\Delta L$ represents the filling ratio of the air, ΔL represents the interval between two branches. The density and bulk modulus of the air and polymer matrix composites (PMC 780) materials are ρ_{air} = 1.204 kg/m^3, ρ_{PMC} = 1020 kg/m^3, B_{air} = 1.4×10^5 Pa, B_{PMC} = 2.817×10^8 Pa, respectively. Based on the assumption that the entire metamaterial can be decomposed into a number of small basic sections, the filling ratio of the air can be calculated in the basic sections of the metamaterial structure. To quantify the increase in the wavenumber of guided waves caused by the medium, the effective refractive index for the acoustic metamaterial is used according to the propagation constant $\beta = k_\theta = n_{\mathrm{eff}}k_{\mathrm{air}}$, while k_θ is the wave-vector component along the tangential direction of the arcuate metamaterial. Then the effective refractive index of the metamaterial can be obtained as:

$$n_{\text{eff}}(N,f)=\sqrt{\frac{\rho_{\text{PMC}}B_{\text{PMC}}}{\rho_\theta B}\tan^2\left(f\pi d(N)\sqrt{\frac{\rho_\theta}{B}}\right)+n_{\text{PMC}}}\,, \quad (4)$$

$$n_{\text{PMC}}=v_{\text{air}}\sqrt{\frac{\rho_{\text{PMC}}}{G_{\text{PMC}}}}\,. \quad (5)$$

where f is the frequency of acoustic waves. ρ_{PMC} and B_{PMC} represent the intensity and bulk modulus of the PMC materials. n_{PMC} is the refractive index of the PMC materials, v_{air} is the velocity of the acoustic wave in the air and G_{PMC} = 563 kPa is the shear modulus of the PMC materials.

Assuming $0.1 < F < 0.9$, because $\rho_{\text{PMC}} >> \rho_{\text{air}}$, $B_{\text{PMC}} >> B_{\text{air}}$, equation 4 can be simplified as:

$$n_{\text{eff}}(N,f)=\sqrt{\frac{\rho_{\text{PMC}}B_{\text{PMC}}}{\rho_\theta B}\tan^2\left(f\pi d(N)\sqrt{F(1-F)\frac{\rho_{\text{PMC}}}{B_{\text{PMC}}}}\right)+n_{\text{PMC}}}\,, \quad (6)$$

According to equation 6, which branch the refractive index reaches its maximum value is mainly determined by $f\pi d(N)\sqrt{F(1-F)\frac{\rho_{\text{PMC}}}{B_{\text{PMC}}}}$.

2. Calculation of displacement distribution in BSEM

The spatial and spectral distributions of displacement fields in metamaterial structures can be denoted as:

$$u(N,f)=A(N,f)e^{ik_{\text{PMC}}\int_0^{L_n} n_{\text{eff}}dL}\,, \quad (7)$$

$$A(N,f)=\frac{(2\pi\rho_{\text{PMC}}f)^{1/2}(1-n_{\text{eff}}^{-2})^{1/4}}{\cos[\arctan(\rho_\theta\rho_{\text{PMC}}^{-1}(n_{\text{eff}}^2-1)^{1/2})]}\,. \quad (8)$$

Here, the integral $\int_0^{L_n} n_{\text{eff}}dL$ is the phase shift of the propagation acoustic wave. The A (N, f) in the equation is a slow-varying coefficient function of the pressure field.

3. Acoustic stimulation of auditory brainstem response

The acoustic and electrical stimulation instrument system and the recording instrument system of auditory brainstem potential response used in this experiment and its settings are shown in **Figure S21**. For Acoustic stimulation, at first, sinusoidal waves generated by a functional generator (GFG-8016G, Good Will Inst Co., Ltd, Bayan Lepas, Penang, Malaysia) were fed into a custom-made burst generator (electronic switch) and 40-ms tone burst (0.5-ms rise and decay times) was formed under the drive of a synchronous signal generator (Master-8, Israel).

Then, the tone burst was fed into a decade attenuator (LAT-45, Leader, Kohokuku, Yokohama, Japan) for adjusting the amplitude of the sound stimulus was successively sent to a custom-made power amplifier for amplification through the output connection of the attenuator while the output of the power amplifier connected a small loudspeaker (AKG model CK 50, 1.5 cm in diameter, frequency response 1-100 kHz) to present sound stimulus to mice at 5 times per second. The loudspeaker was calibrated with a 1/4-inch microphone (4939, B&K, Denmark) placed at the mouse's ear using a measuring amplifier (2610, B&K, Denmark). The output of the loudspeaker was expressed in decibel sound pressure level (dB SPL) in reference to 20 mPa root mean square. A frequency response curve of the loudspeaker was plotted to determine the maximal available sound amplitude at each frequency. The maximal stimulus level ranged from 95 to 120 dB SPL between 10 and 80 kHz but dropped off sharply to 80 dB SPL at 100 kHz thereafter. According to the acoustic sensitive frequency range of human audiogram, six different acoustic stimulus frequencies (300 Hz, 500 Hz, 1000 Hz, 2000 Hz, 3000 Hz, 5000 Hz) were used in this experiment.

4. Electric stimulation of auditory brainstem response

In this experiment, a pair of electrical stimulation electrodes, one of which was an insulated tapered tungsten electrode and was inserted into the cochlea through a small hole prearranged near the round window to stimulate the cochlea; the other of which was a silver wire electrode as a reference electrode insert and was fixed under the scalp at the surgical site where the auditory bulla was exposed. Two types of electrical stimulation were used in the experiment. The first type of electrical stimulation is the electrical signal generated by an acoustic-electrical conversion of BSEM, which is amplified by the power amplifier and used to trigger the electronic stimulator to output different amplitudes of square wave electrical signals to stimulate the cochlea of mice. The preparation method is as follows: tone burst is formed as described in the acoustic stimulation method above. Sound wave vibration generated by a loudspeaker (Φ3 cm, 4Ω, 20-20000 Hz) (Simaier Ltd, China) was transmitted to BSEM to produce acoustic-electric conversion through a plastic rod with the length of 40 mm length and diameter of 5 mm fixed on the loudspeaker, and then generated electric signals during acoustic-electric conversion were fed into a custom-made power amplifier for amplification and were used to trigger stimulator to output square wave signal stimulating the cochlea of mice. The second type of electrical stimulation is to stimulate directly the cochlea of mice with the electric signal generated by acoustic-electric conversion from BSEM.

5. Recording of evoked auditory brainstem responses

In this experiment, the auditory brainstem potential responses evoked by sound and electrical stimuli were recorded using stainless steel needle-like dual recording electrodes, one of which was inserted under the scalp directly opposite the intersection of sagittal and lambdoidal sutures and the other under the scalp directly opposite the intersection of sagittal and coronal sutures on the mouse skull. The two electrodes are wired to the input of the probe connected with a preamplifier (ISO-DAM, WPI, Sarasota, FL, USA) and filtered by a bandpass filter (Krohn-Hite 3500, Oceanside, CA, USA). Then, the recorded potentials were respectively led into an oscilloscope (TDS210, Tek, Beaverton, OR, United States) for visual monitoring and an analogue-to-digital converter equipped with a data acquisition system (Digidata 1322A, Axon, USA) for sampling, storage, and subsequent processing. Auditory brainstem evoked potentials were sampled 300 times and averaged using PCLAMP 8.1 software (Axon Instrument, USA). This process was repeated more than 10 times under each stimulation condition to ensure the accuracy and stability of potential recording.

6. Results of ABR under electrical stimulations

The results of ABR under acoustic stimulations are shown in **Figure S24**. The results for different frequencies and amplitudes are similar to previous studies on human and animals.[32, 37, 38] The amplitude of acoustic stimulus has an obvious influence on evoked potential response, and the response amplitude decreases with the decrease of acoustic stimuli. These results suggest that the number of cochlear nerve fibers excited by acoustic stimulation and the input of nerve impulses are both reduced with the amplitude decrease of acoustic stimulation. It is essentially a manifestation of a basic neural response characteristic, and its mechanism is basically the same as the physiological mechanism of hearing. In order to verify the role of the tympanic membrane and ossicular chain in acoustic conduction, no response of the auditory brainstem to acoustic stimulation was observed from the scanning results after the experimental destruction of the tympanic membrane and ossicular chain of both ears. This is due to the interruption of sound wave transmission to the inner ear. The results of statistical analysis showed that the amplitude of auditory brainstem evoked potentials decreased with the decrease of the amplitude of acoustic stimulation, which showed a consistent trend, indicating that the characteristics of auditory brainstem evoked potentials were determined by theirphysiological characteristics. These results indicate that auditory brainstem evoked potentials can be induced by acoustic stimulation in mice, and the auditory system has good frequency response and hearing ability.

7. Results of ABR under electrical stimulations

The supplementary results (different excitation intensities, 85 dB and 80 dB) of ABR under direct electrical stimulations at different frequencies by BSEM are illustrated in **Figure S22**. The results of ABR under electrical stimulations by BSEM with standard stimulators are depicted in **Figure S25**. It should be noted that the overall change tendency of the maximum peak of the potential response decreased with the increase of the sound frequency calculated according to square wave frequency.

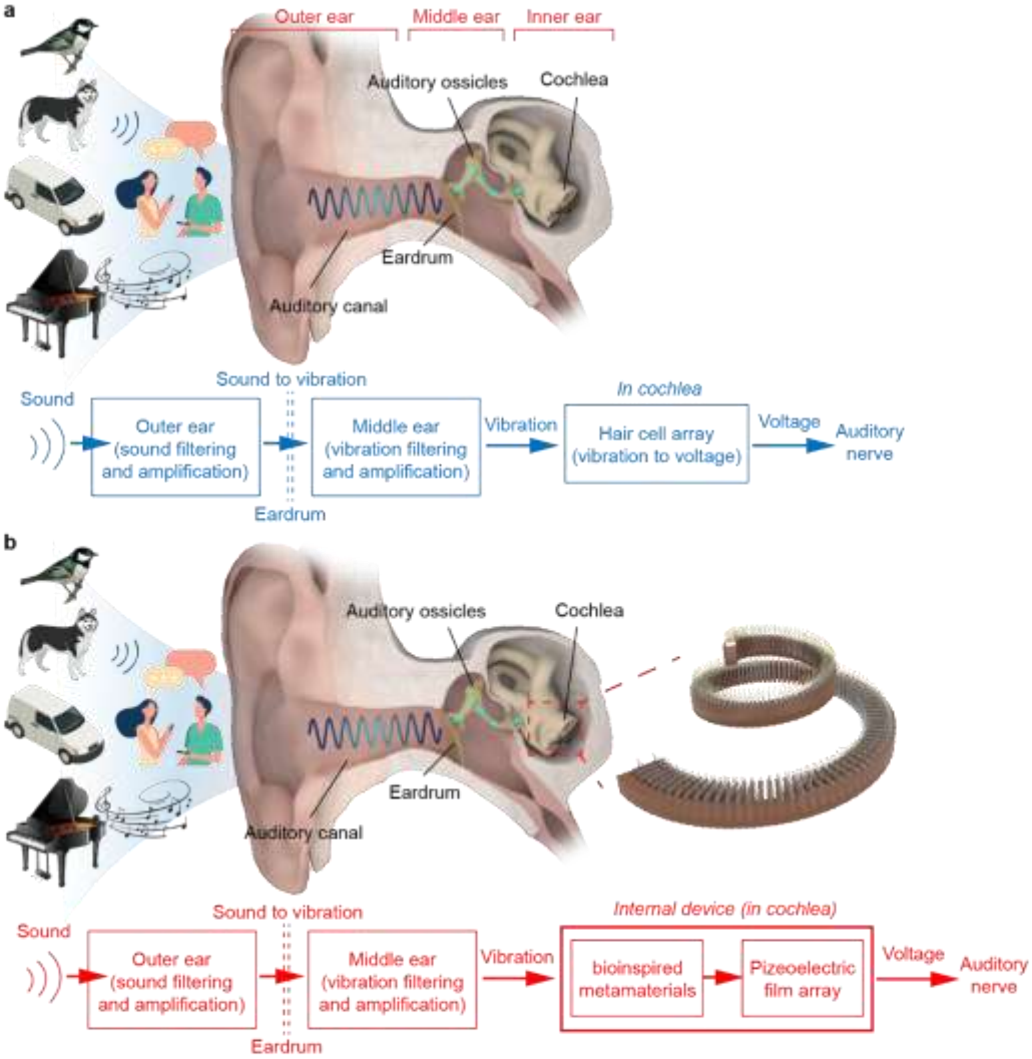

Figure S1. Hearing process of normal people **(a)** and BSEM users **(b)**.

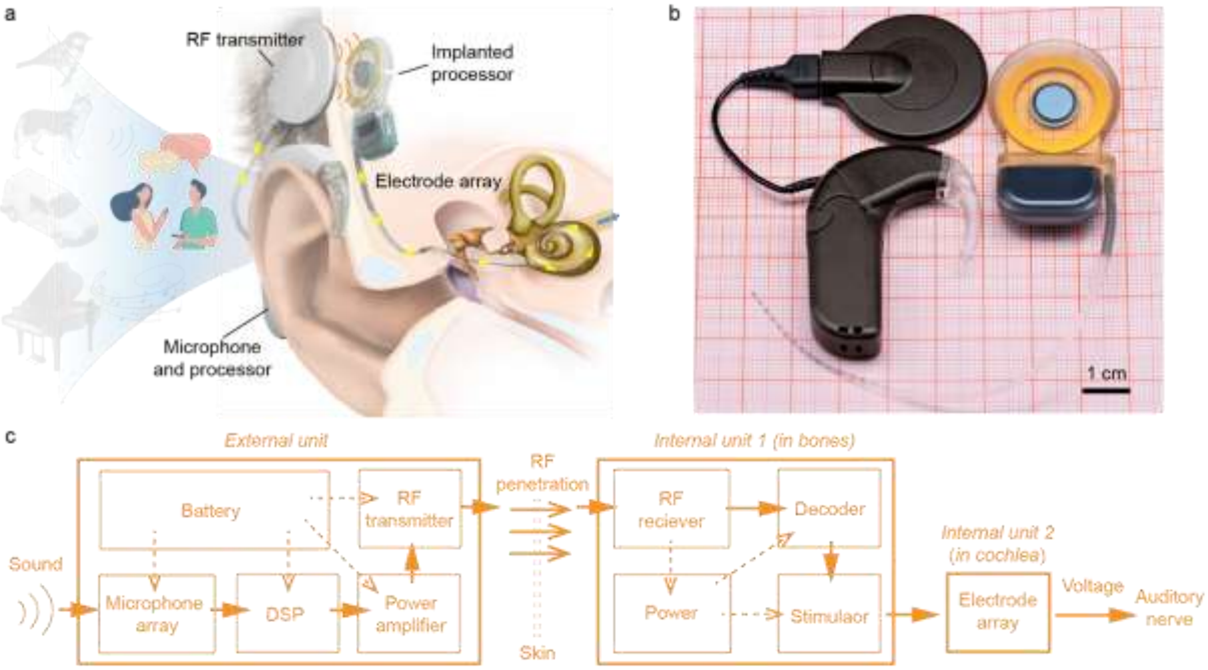

Figure S2. a) Hearing process of cochlear implants (CI) users. The image is modified from the website (https://www.cochlear.com). **b)** A photograph of the commercial CI. **c)** Block Diagram of the component composition of a typical CI.

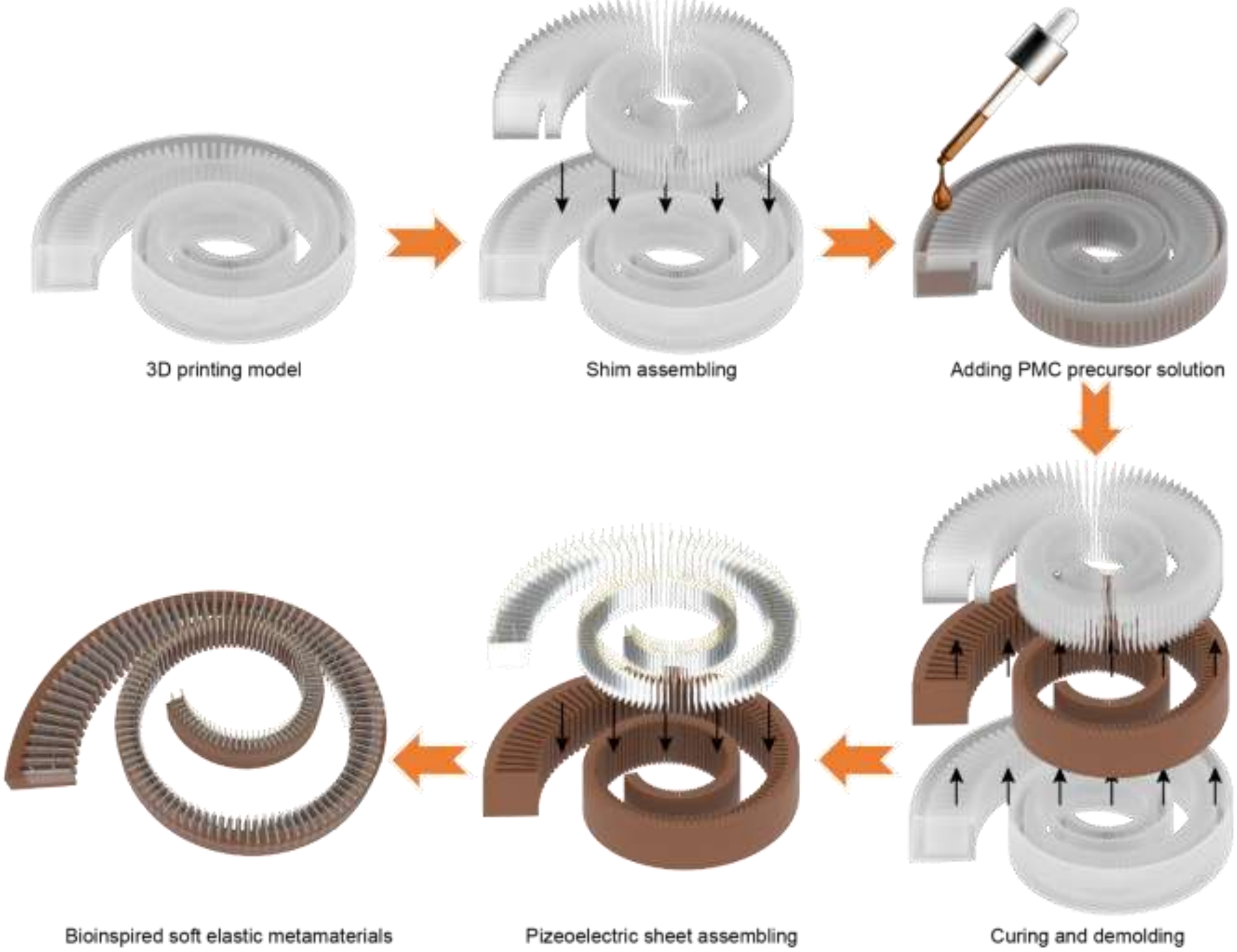

Figure S3. The fabrication process of BSEM.

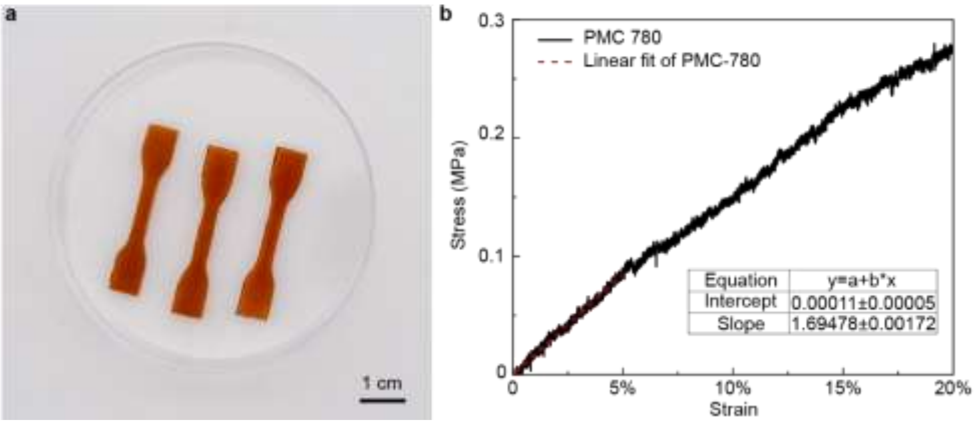

Figure S4. Uniaxial tensile tests of PMC 780. (A) Test samples. (B) Strain-stress curve of PMC-780.

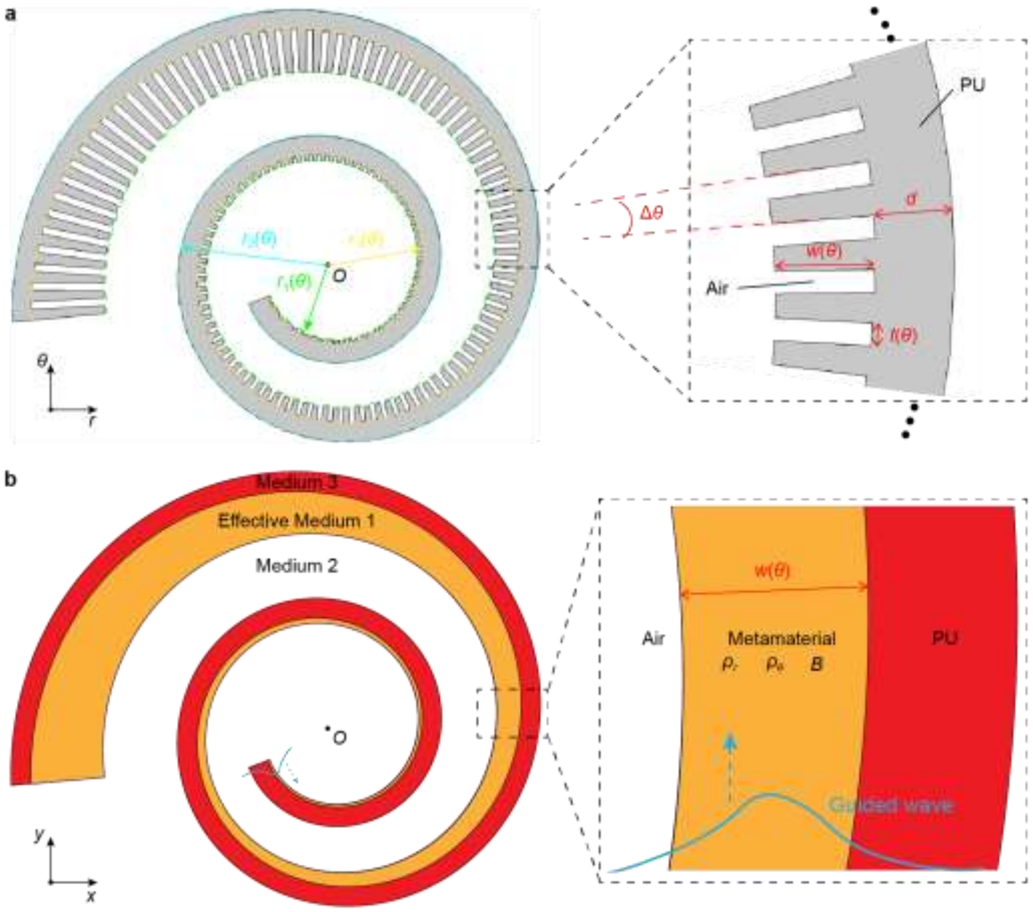

Figure S5. a) Structural parameters of the bioinspired soft elastic metamaterial. **b**) Effective model of the bioinspired soft elastic metamaterial.

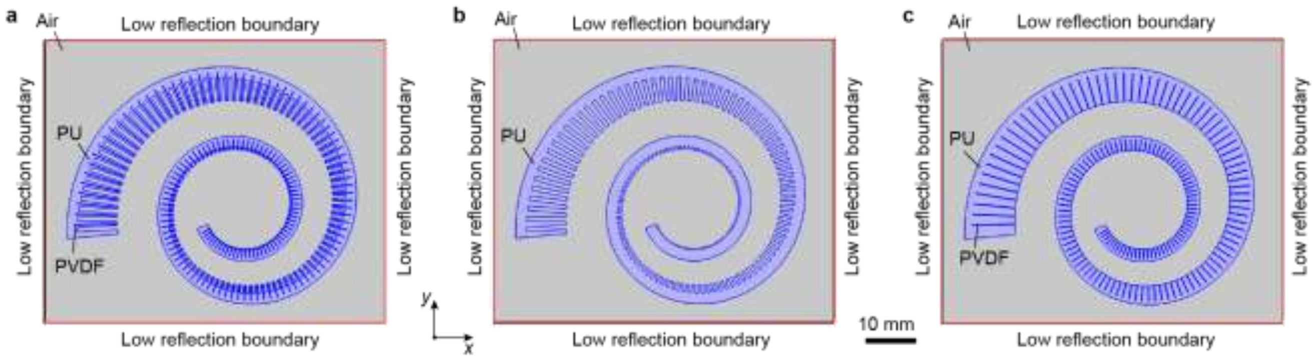

Figure S6. Simulation model and conditions of BSEM with piezoelectric films (a), BSEM (b), and unstructured matrix with piezoelectric flakes (c). Low reflection boundary conditions are added around the simulation models.

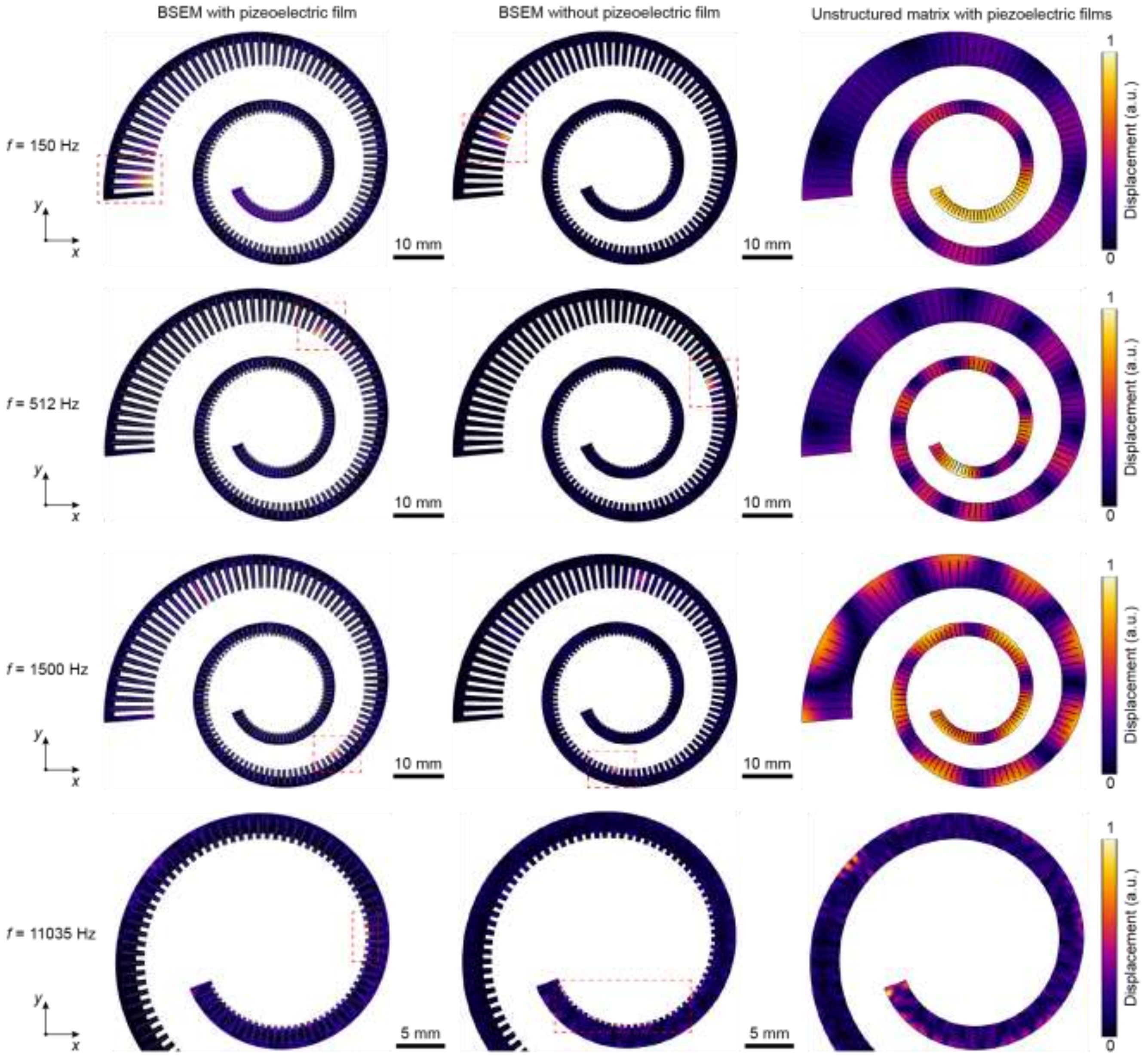

Figure S7. Simulated displacement distribution at different frequencies (vertical) for BSEM with piezoelectric films, BSEM, and unstructured matrix with piezoelectric films (horizontal).

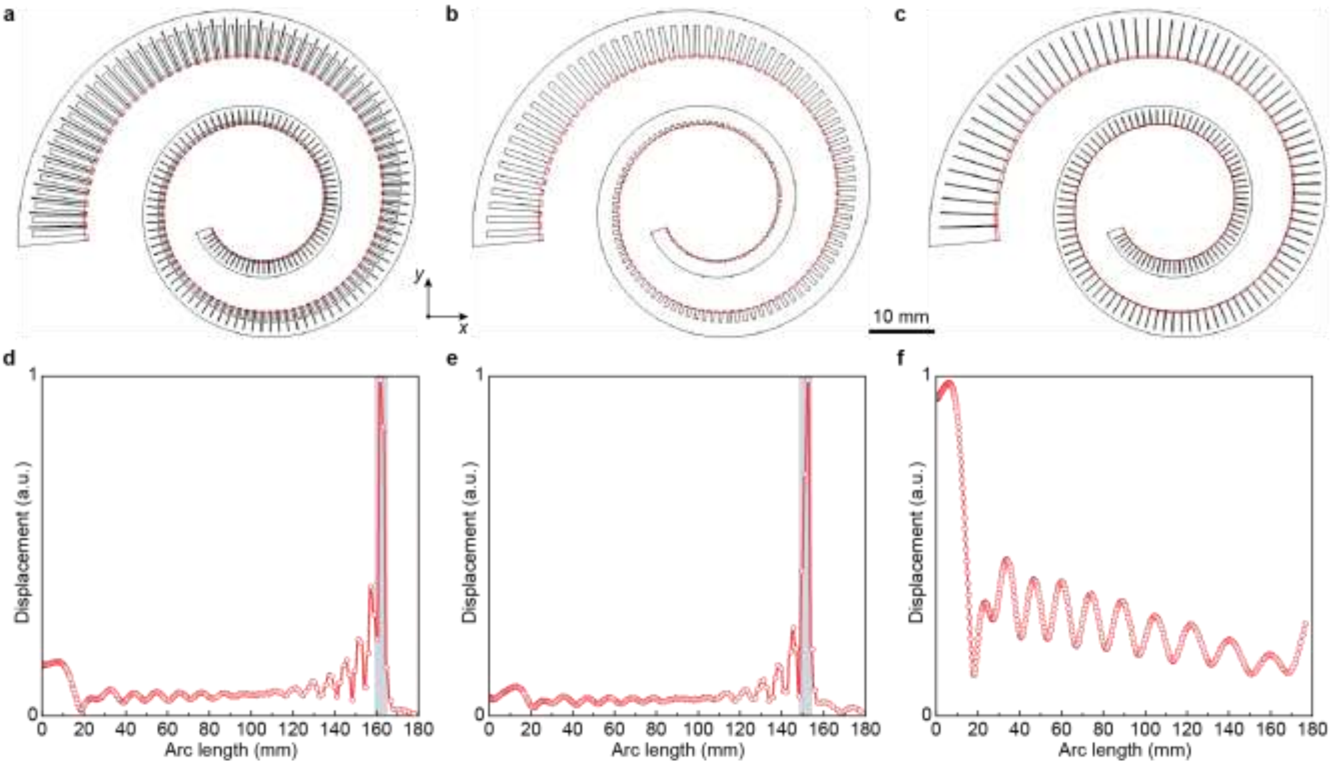

Figure S8. a-c) Sketch for selected profiles of BSEM with piezoelectric films, BSEM, and unstructured matrix with piezoelectric flakes, respectively. d-f) Displacement distributions on profiles shown in (a-c).

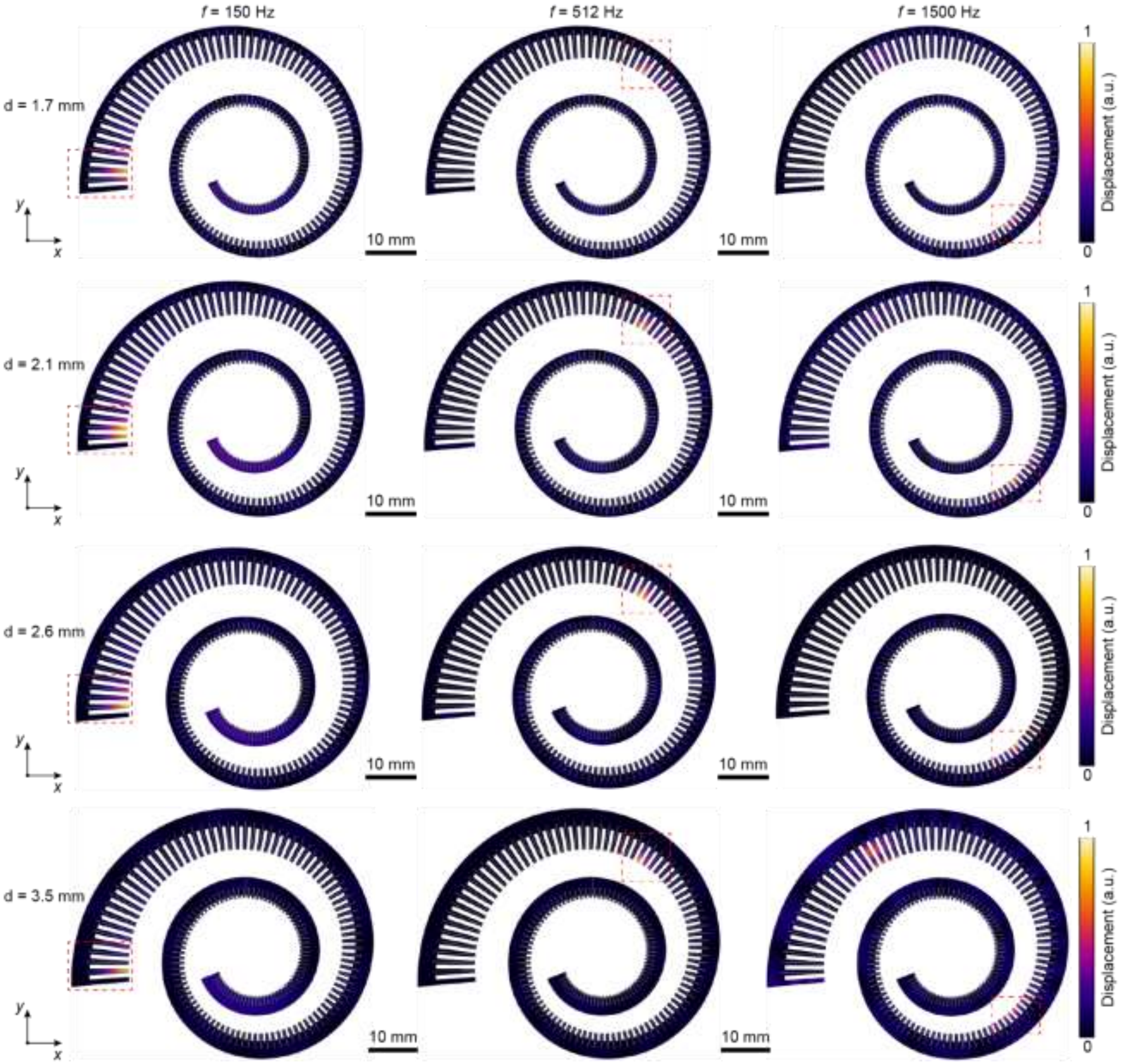

Figure S9. The influence of bottom margin *d* to BSEM. Vertical: different *d*. Horizontal: different frequencies.

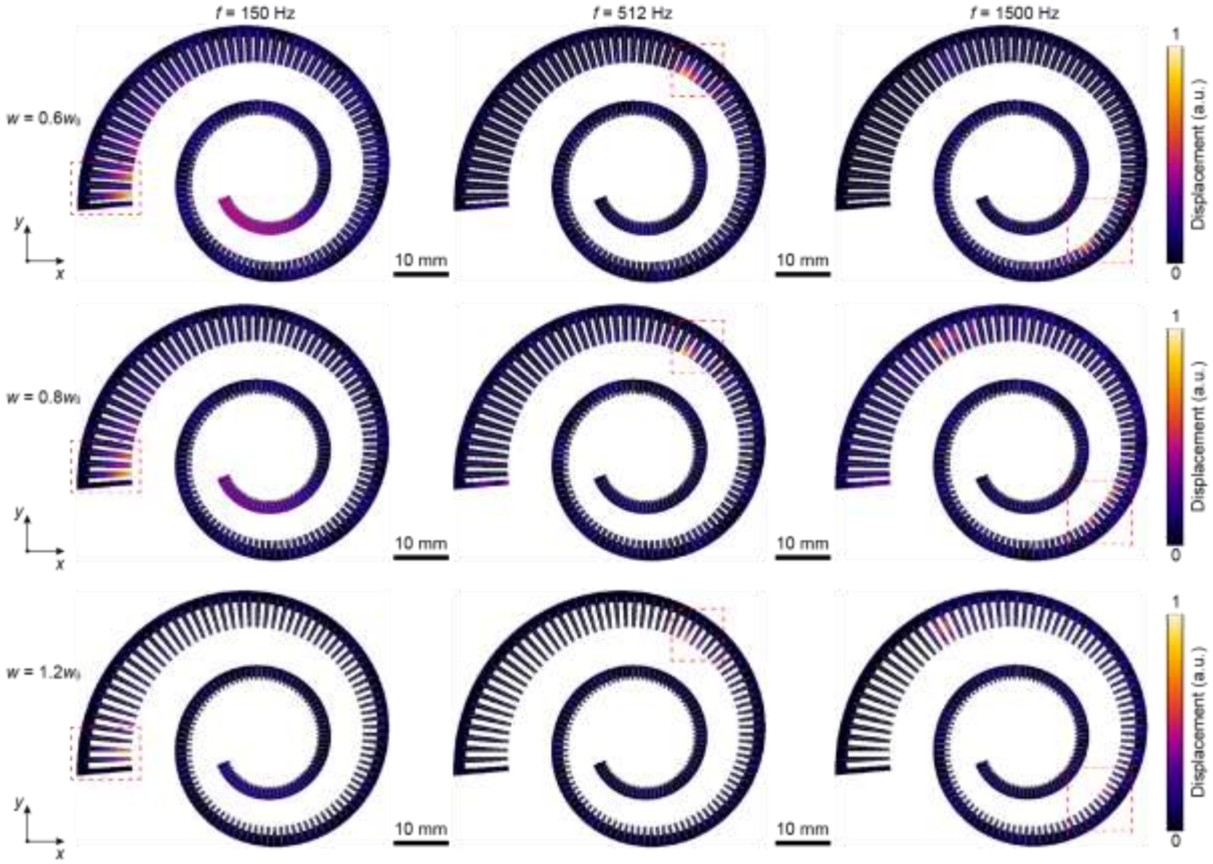

Figure S10. The influence of cut cuboid width *w* to BSEM. w_0 represent the width utilized in the Maintext. Vertical: different width *w*. Horizontal: different frequencies.

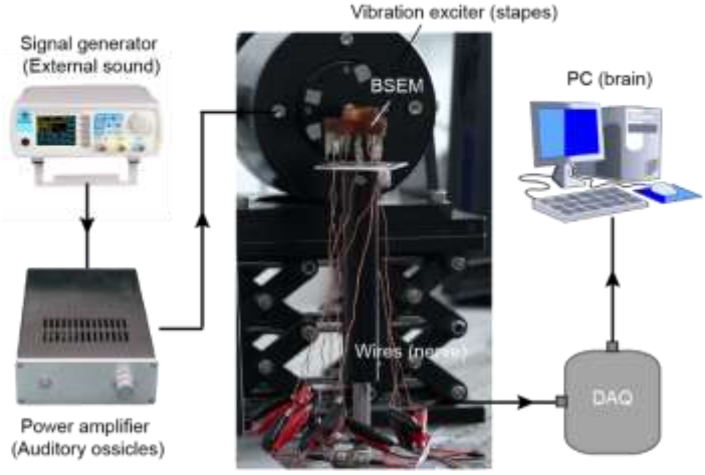

Figure S11. Experimental setup for measuring output voltages of BSEM.

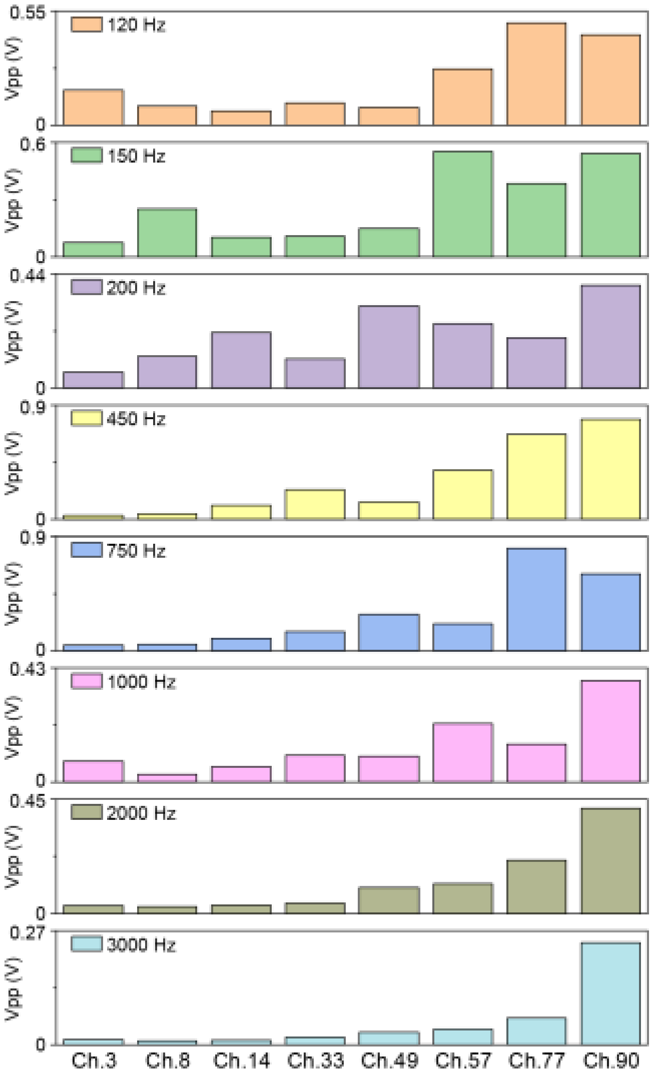

Figure S12. Peak-to-peak voltage (Vpp) of other channels in BSEM. The results show a considerable frequency range (120-3000 Hz) of BSEM.

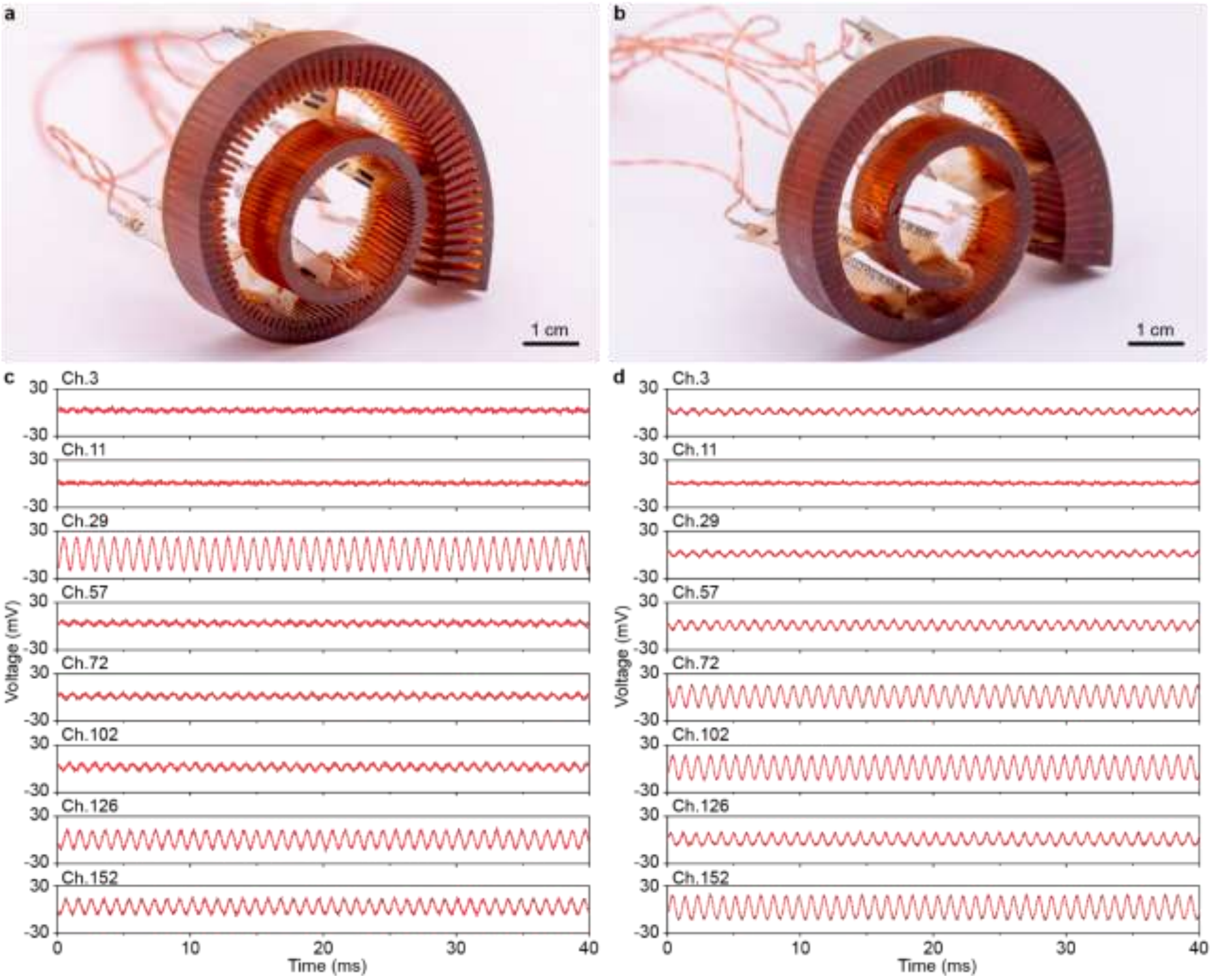

Figure S13. Output voltage measurements of BSEM and unstructured matrix with piezoelectric flakes (control group). a, b) Photographs of BSEM and control group, respectively. c, d) acquired voltages (8 channels) of BSEM and the control group at 420 Hz, respectively.

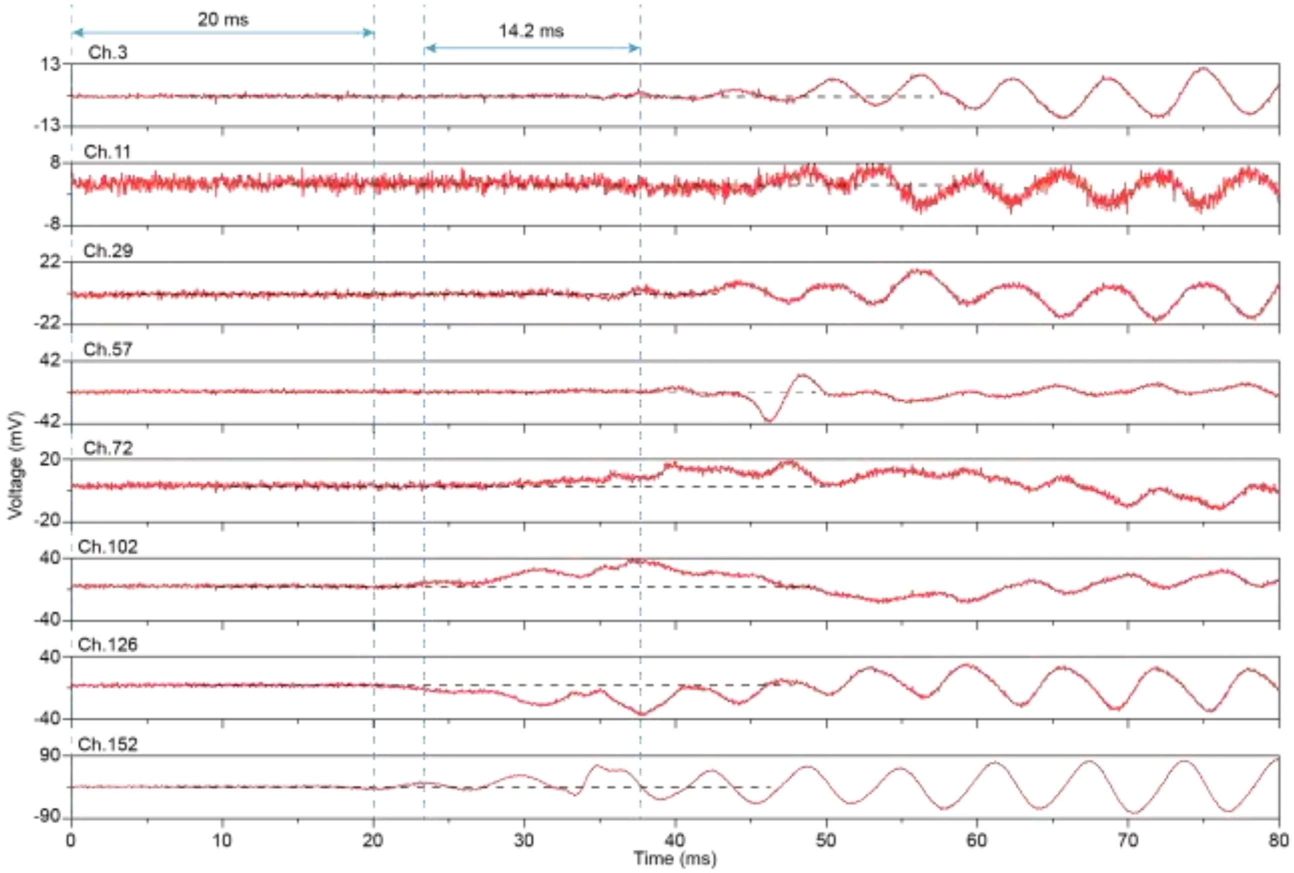

Figure S14. Comparison of signal generation time of 8 channels in BSEM under single short-time excitation.

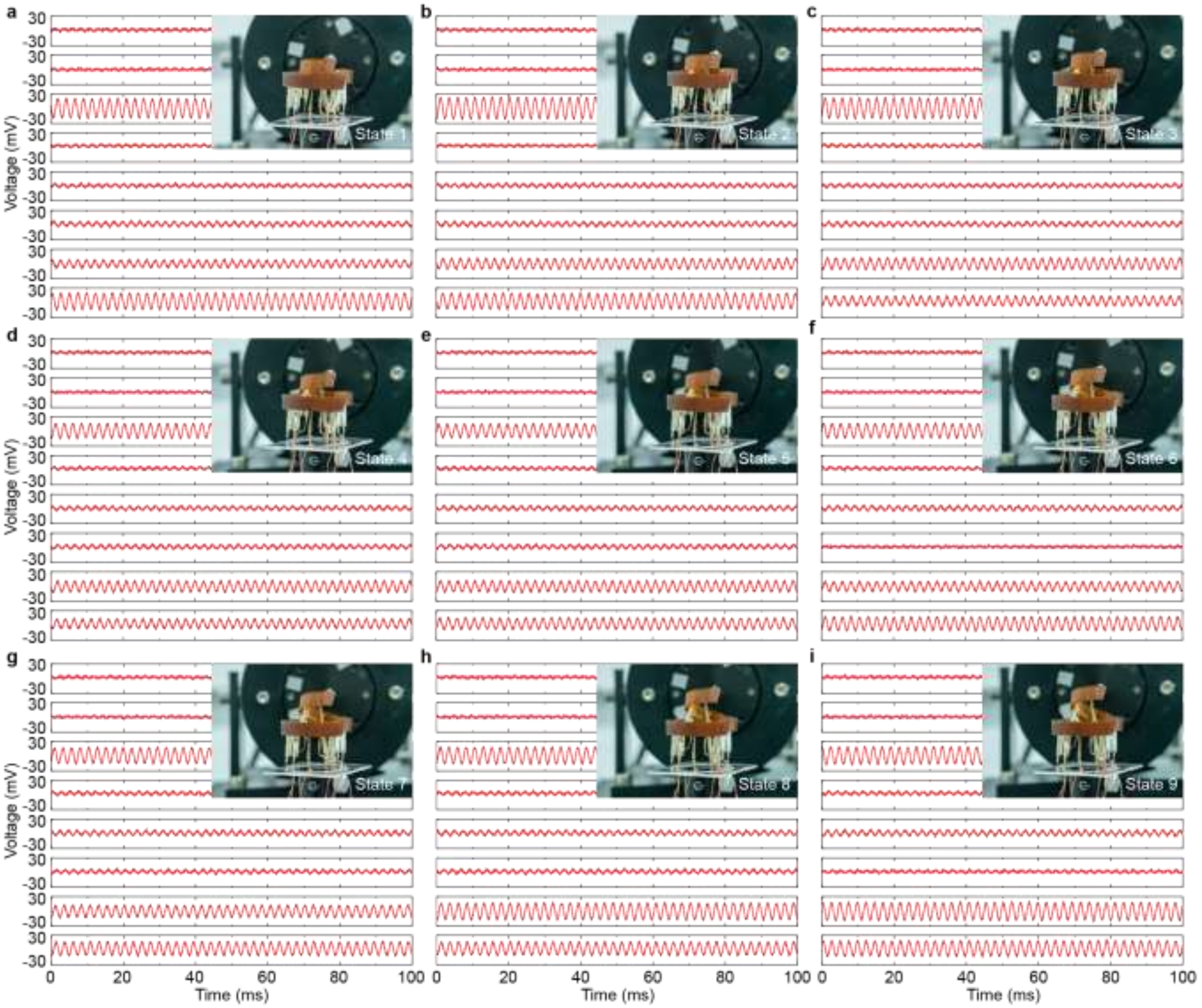

Figure S15. Comparison of signal generation time of 8 channels in BSEM. a-i) Output voltages (8 channels) of BSEM under the stretch of different levels from original to stretched by 5 cm, respectively. Output voltages nearly remain the same when BSEM was stretched.

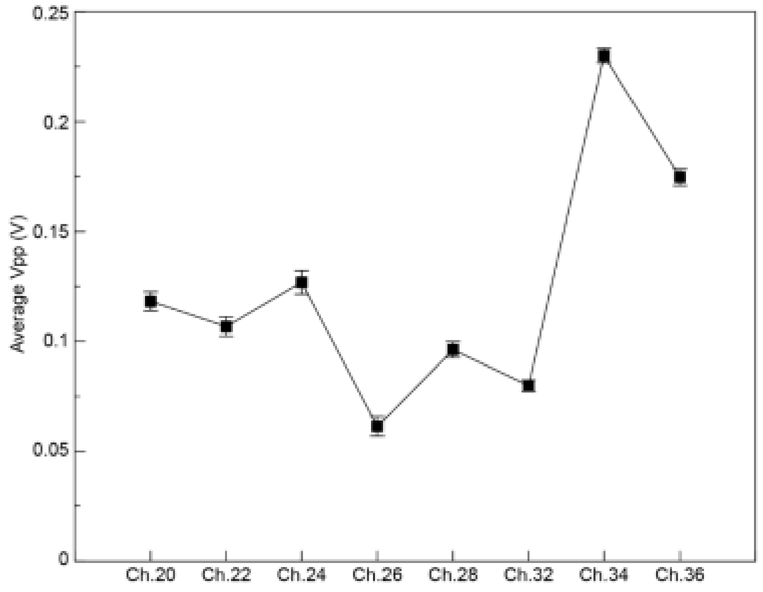

Figure S16. Average V_{pp} of 8 channels under 300 times tone-burst excitation. The center frequency and duration of each tone-burst are 493 Hz and 40 ms. respectively.

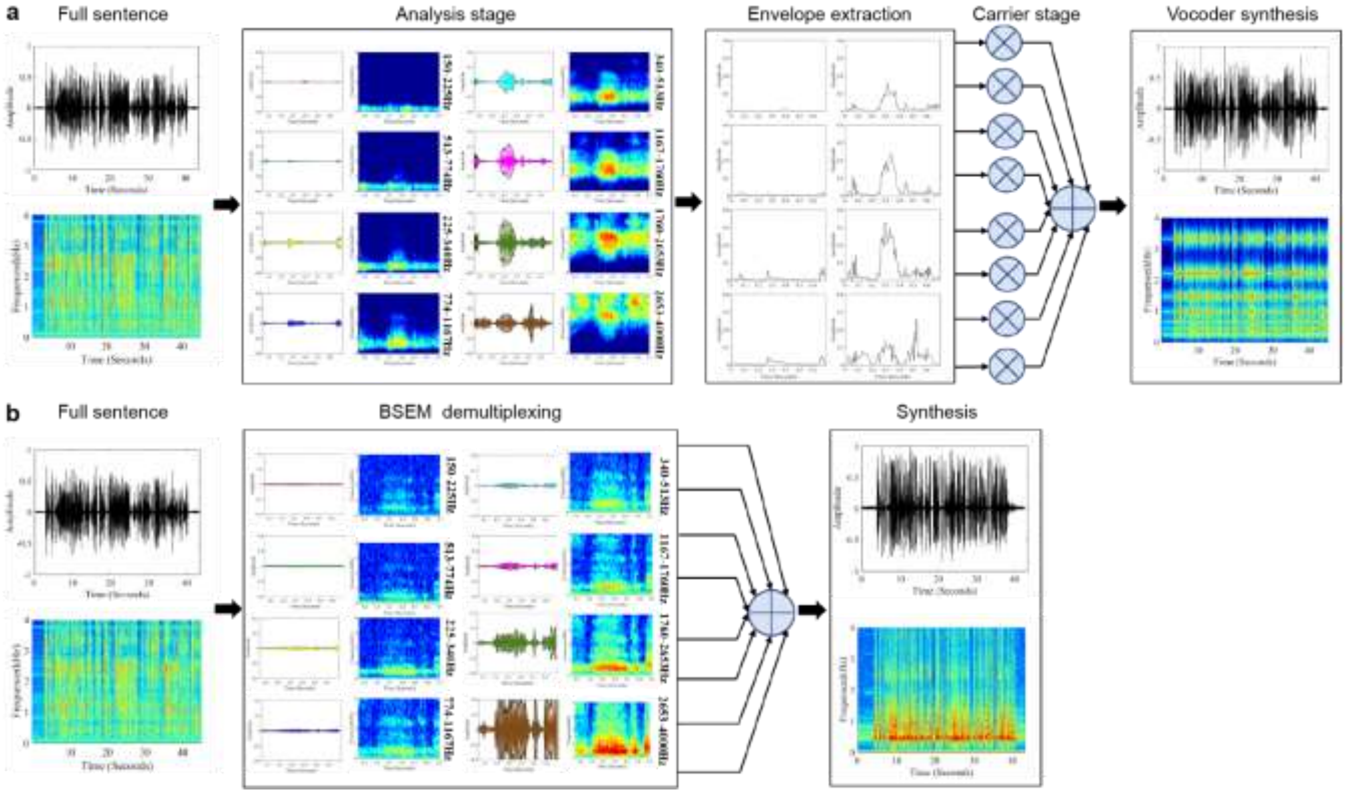

Figure S17. The processing methods of CI (a) and BSEM (b).

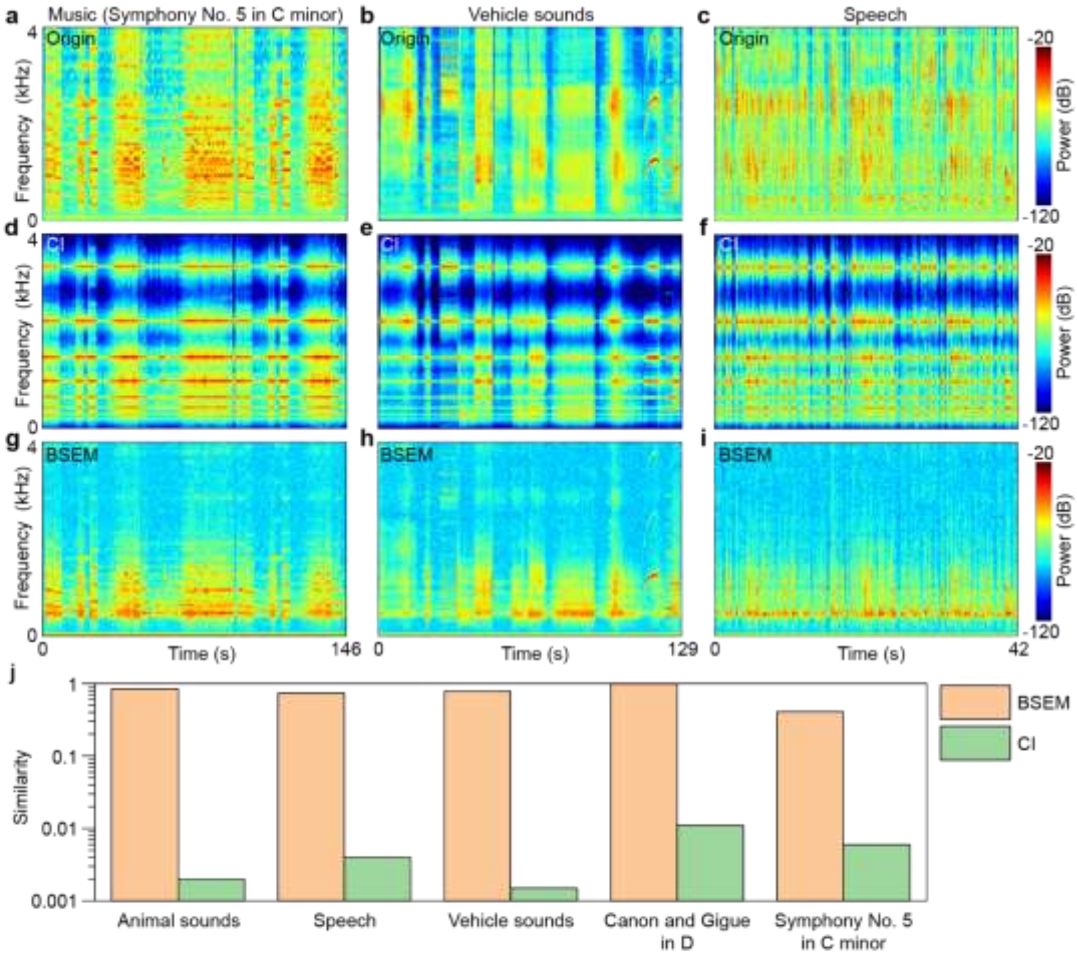

Figure S18. Spectrograms of different sounds processed by CI and BSEM. a-c) Original spectrograms of a symphony, vehicle sounds, and a speech, respectively. The frequency range is limited to 0-4 kHz. d-f) Spectrograms (processed by an 8-channel CI, processing methods are shown in Figure S17) of music, vehicle sounds, and a speech, respectively. g-i) Spectrograms (processed by 8 channels of BSEM) of music, vehicle sounds, and a speech, respectively. j) Similarity comparison of sounds processed by BSEM and CI. Similarity calculation based on the spectrograms of different sounds by a software (Sound-Similar, https://www.virtins.com/VT-Sound-Recognition.shtml)

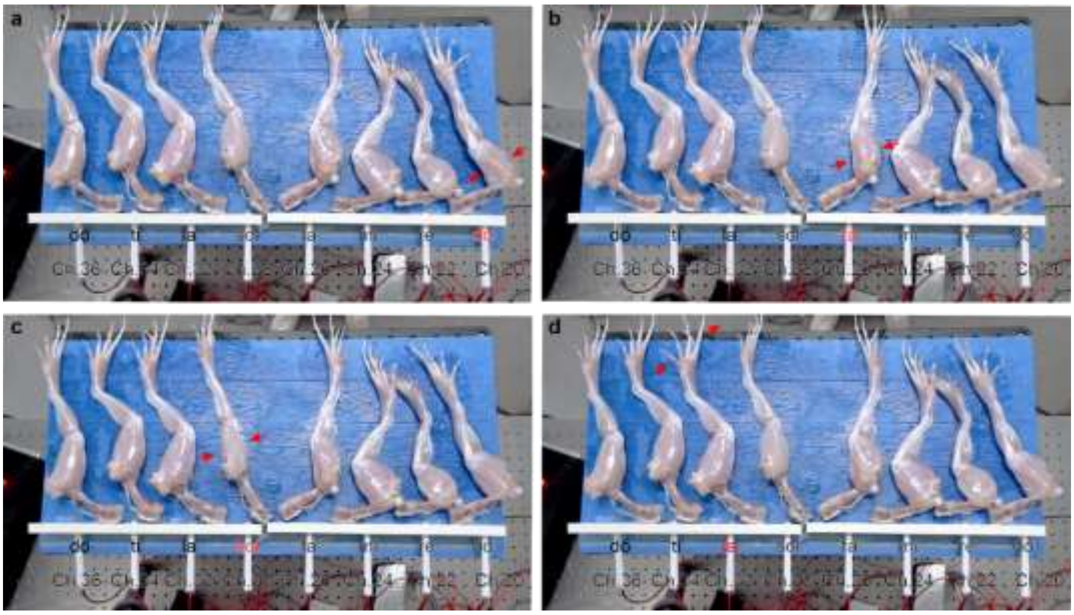

Figure S19. Frog leg bouncing when stimulated by 8 channels of BSEM at excitations of piano-key tones in C major scale. a-d) Photographs of frog leg bouncing at excitations of “do”, “fa”, “sol”, and “la”, respectively.

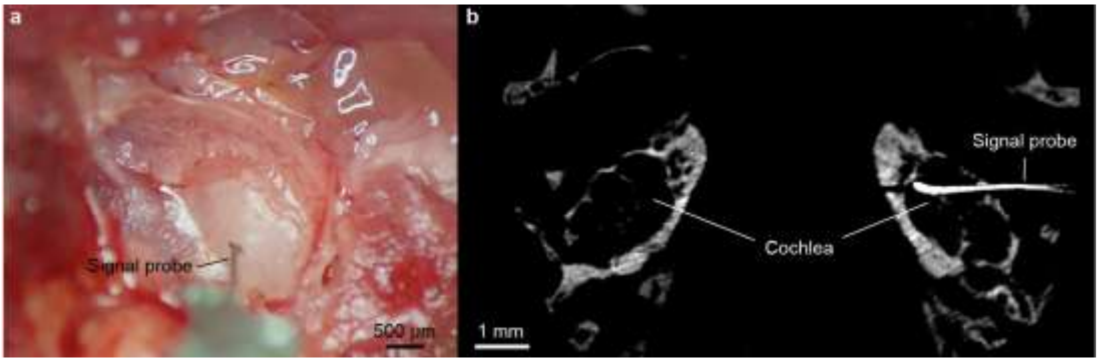

Figure S20. Optical (a) and micro CT (b) image of electrode insertion position in mice cochlea.

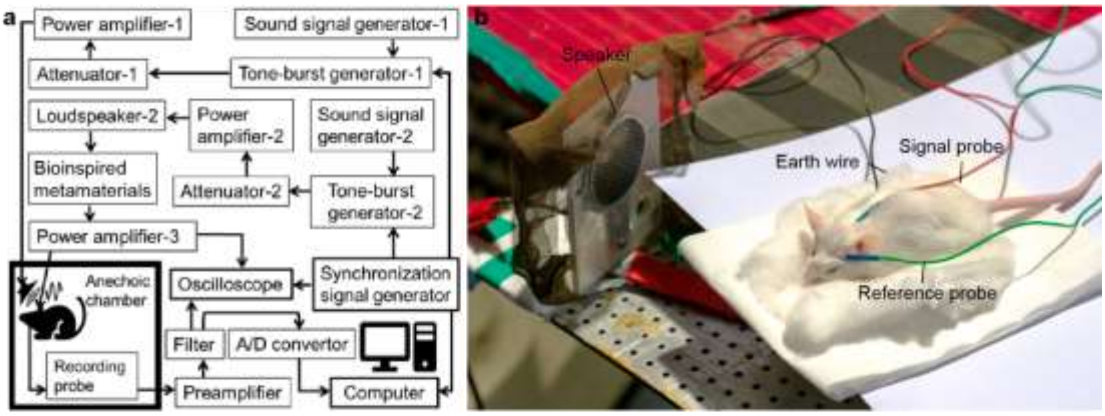

Figure S21. Experimental set-up for ABR acquisition of mice. **a**) Sketch of set-ups for animal experiments on mice. Numbers 1, 2 and 3 denote the devices used in acoustic stimulations, direct electric stimulations by BSEM, and electric stimulations by standard stimulators (with power amplifier), respectively. **b**) Photograph of a mouse under acoustic stimulations.

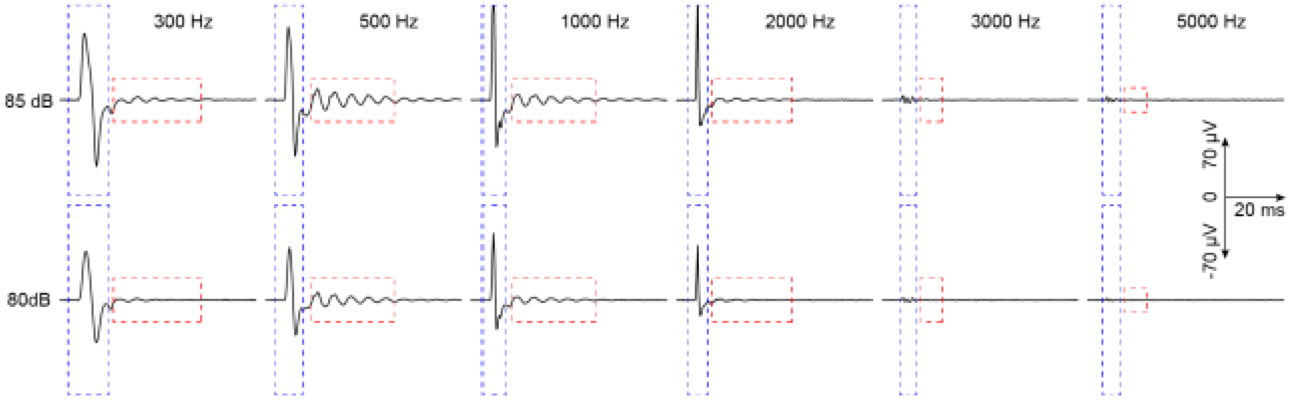

Figure S22. ABR signals of mice under electrical stimulation by BSEM at different excitation intensities. The right angle indicates the scale of duration and amplitude of the potential response in the whole figure.

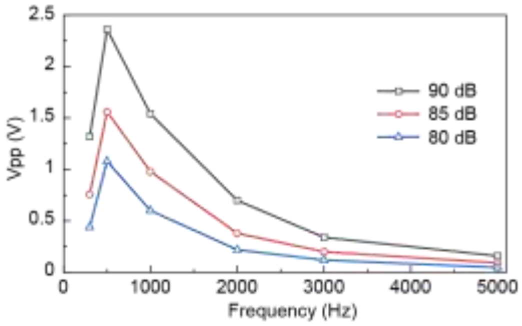

Figure S23. Output voltage of BSEM for each frequency in ABR acquisition experiments.

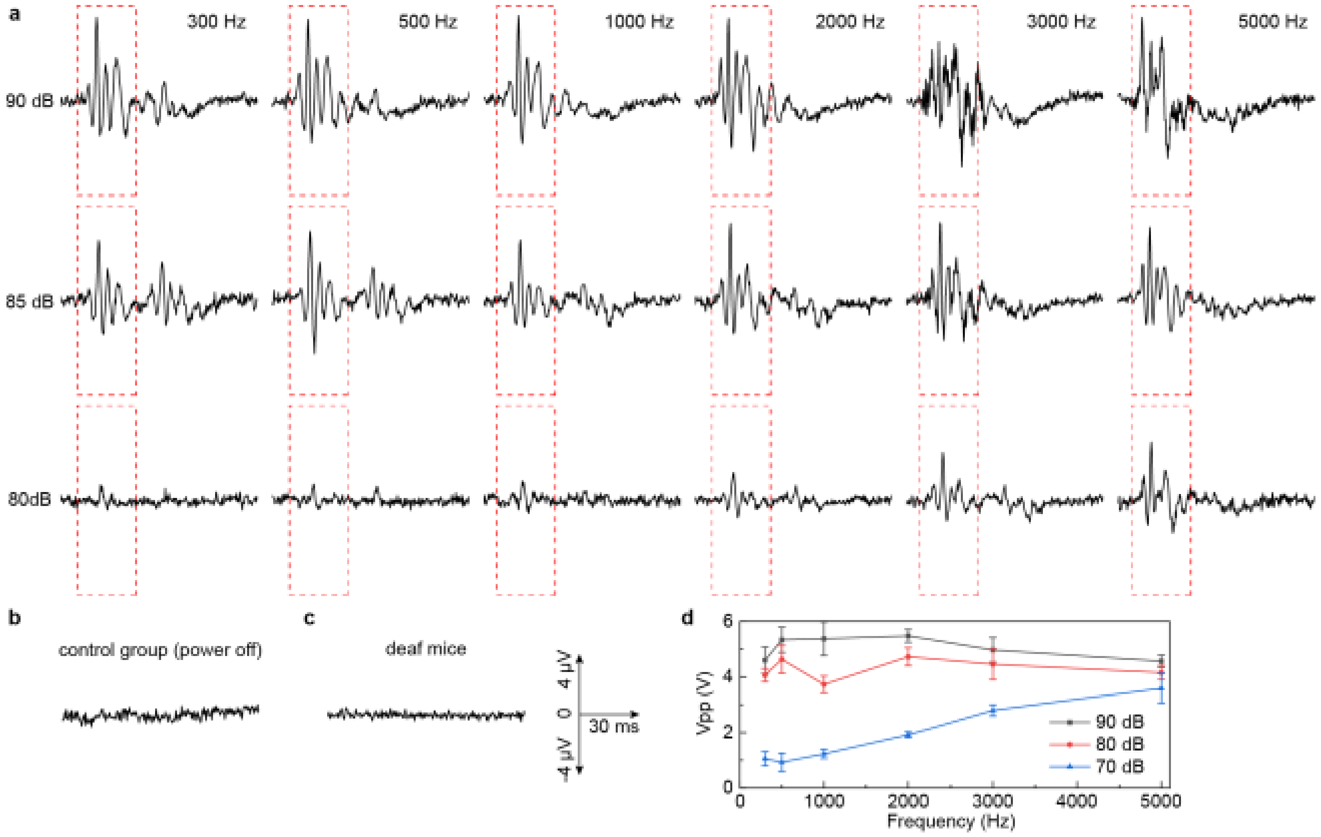

Figure S24. ABR signals of mice under acoustic stimulations at different frequencies. **a**) The responses to tone bursts of 6 frequencies and 3 amplitudes with 20-ms duration. **b**) Scan-sampling results obtained without any acoustic stimulation (control). **c**) Results obtained on deaf mice. The right angle indicates the scale of duration and amplitude of the potential response in the whole figure. **d**) The statistical results of ABR under acoustic stimulations at different frequencies. Each sampling is the summation and average of the evoked potentials induced by 300 electrical stimulation of cochlea under each same stimulation condition, and the results were repeated 10 times. The peak-to-peak average of the maximum wave of the evoked potential reaction is then calculated, with the positive and negative vertical bars at each data point being the standard deviation (i.e. ±SD).

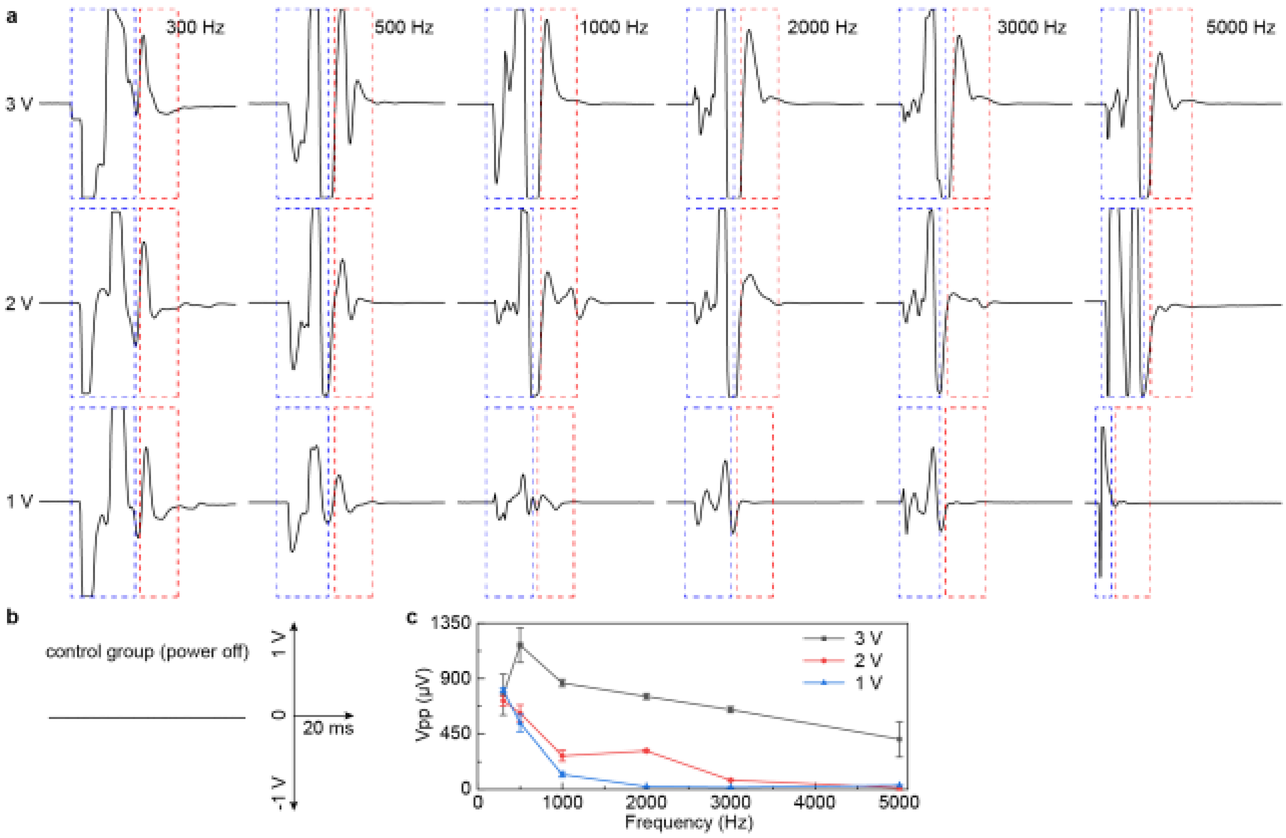

Figure S25. ABR signals of mice under electrical stimulation by BSEM with stimulators at different frequencies. **a**) ABR induced by unilateral cochlea stimulation of square wave electrical signals with different frequencies and intensities. Note that the potential waves in the rectangular boxes on each scan result are electrical stimulus artifacts. **b**) The result obtained when the cochlea was not stimulated at all (control). The right angle indicates the scale of the duration and amplitude of ABR in the whole figure. **c**) Results obtained on deaf mice. The right angle indicates the scale of duration and amplitude of the potential response in the whole figure. **d**) The statistical results of ABR at different frequencies. Each sampling is the summation and average of the evoked potentials induced by 300 electrical stimulation of cochlea under each same stimulation condition, and the results were repeated 10 times (n = 10). The peak-to-peak average of the maximum wave of the evoked potential reaction is then calculated, with the positive and negative vertical bars at each data point being the standard deviation (i.e. ±SD).

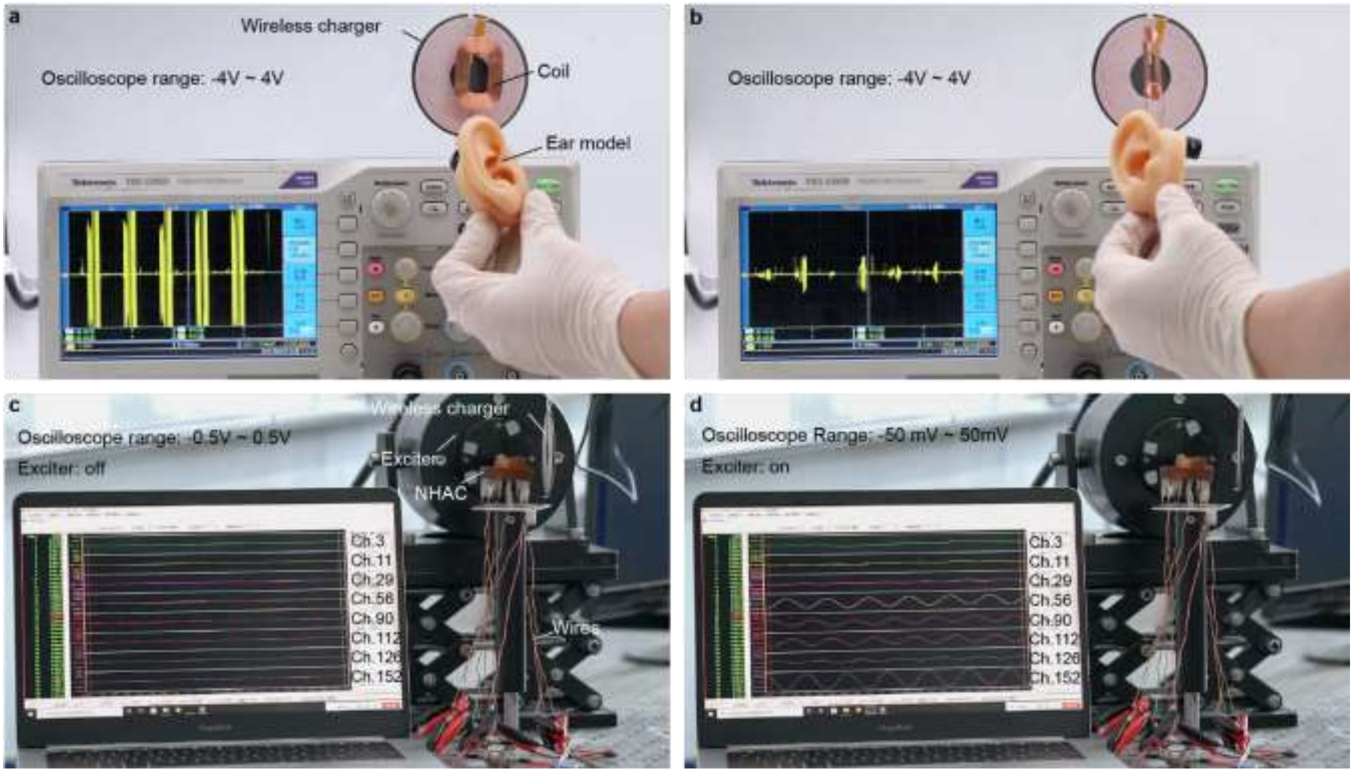

Figure S26. Electromagnetic interference (EMI) tests of a coil (a component in CI) and BSEM. **a, b**) Two states of EMI tests for a coil. Interference voltages are greater than 2 V. **c, d**) Two states of EMI tests for BSEM. Interference voltages are smaller than 10 mV.

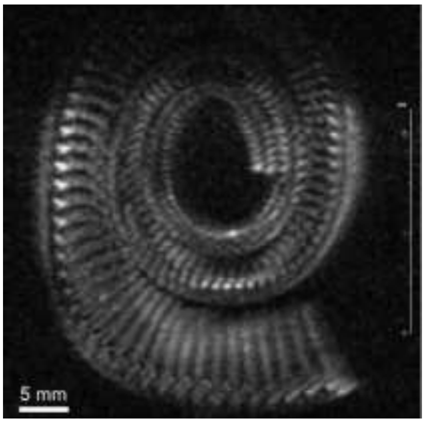

Figure S27. MRI image of BSEM. BSEM is completely compatible with MRI and will not influence the imaging of surrounding tissue.

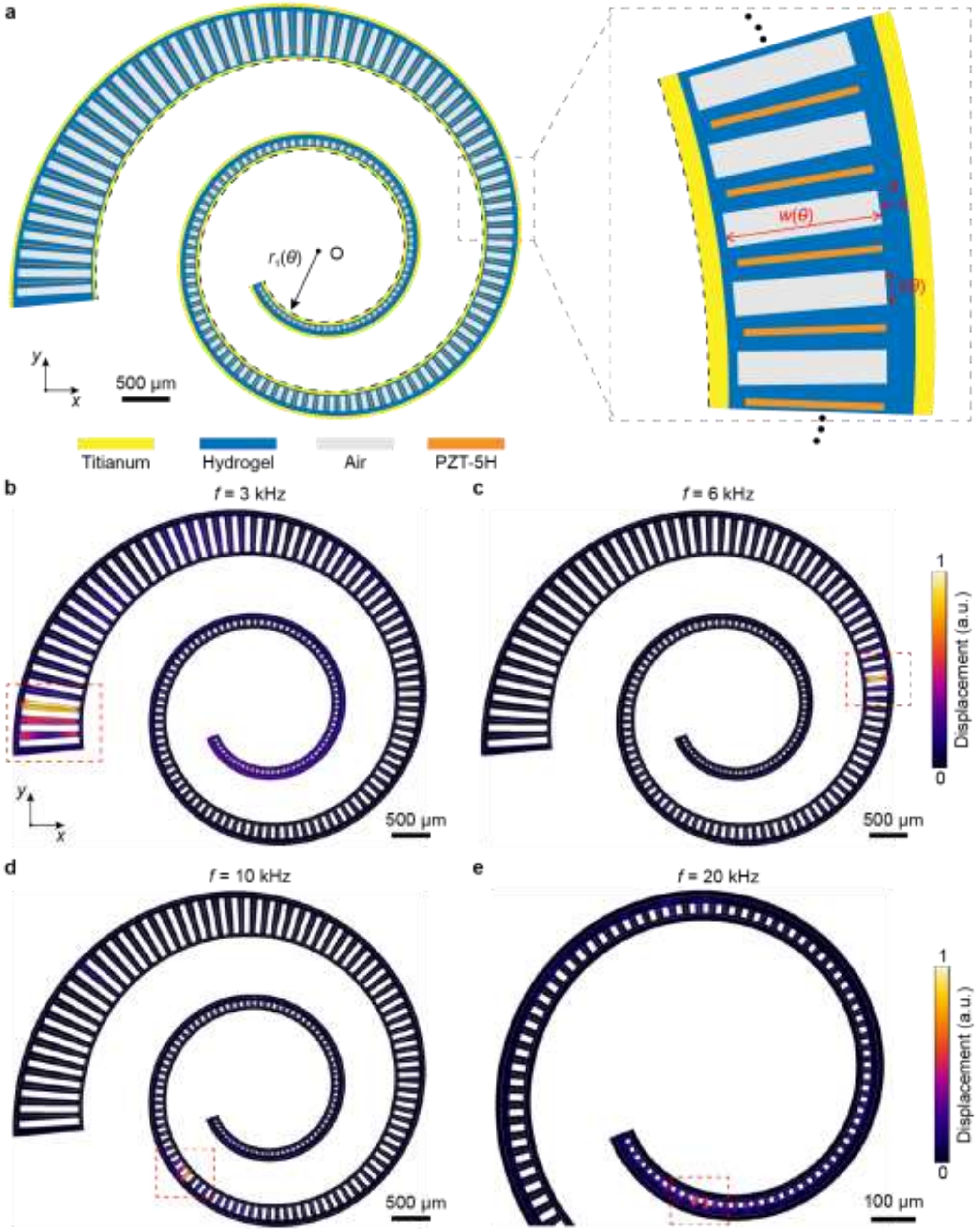

Figure S28. Miniaturization design for BSEM. **a**) Sketch of miniaturized BSEM. **b-d**) Simulated displacement distributions at incident waves at 3 kHz, 6 kHz 10 kHz, and 20 kHz, respectively.

Table S1.
Summary and comparison of various mechanisms for rehabilitating hearing (Aspects of natural hearing).

**Mechanism**	**Signal component**	**Number of channels**	**Number of simultaneous working channels**	**Processing delay**	**Sound source**	Voltage gain	Flexibility	**Examples**
Cochlear Implant	Temporal envelope	24<	1-4	>0.5 s	External environment	/	None	Naida CI Q90, AB
Totally implantable cochlear Implant	Temporal envelope	24<	1-4	>0.5 s	Subcutaneous or tympanic membrane	/	None	Ref. (*7*, *8*)
Rigid resonance plates	/	10<	Unlimited	Non-natural*1	/*[1]	/	None	Ref. (*9-11*)
**BSEM**	**Temporal envelope and temporal fine structure**	**>160**	**Unlimited**	**Without extra delay (Natural delay)**	**Stapes (vibrations)**	**10 times**	**Stretchable and bendable**	**This work**

[1] Rigid resonance plates obtained sound directly from the air; thus, it is difficult to evaluate their sound source in the human body. Besides, every channel in rigid resonance plates receives sound at the same time, which is not in accord with the order that hair cells are activated during the natural hearing.
